# Transcriptome-wide association study-derived genes as potential visceral adipose tissue-specific targets for type 2 diabetes

**DOI:** 10.1007/s00125-023-05978-5

**Published:** 2023-08-04

**Authors:** Haibo Tang, Jie Wang, Peizhi Deng, Yalan Li, Yaoquan Cao, Bo Yi, Liyong Zhu, Shaihong Zhu, Yao Lu

**Affiliations:** 1 Department of Metabolic and Bariatric Surgery, The Third Xiangya Hospital, Central South University, Changsha, China; 2 Clinical Research Center, The Third Xiangya Hospital, Central South University, Changsha, China; 3 School of Life Course Sciences, King’s College London, London, UK

**Keywords:** Candidate genes, Causal inference, Mendelian randomisation, Transcriptome-wide association study, Type 2 diabetes, Visceral adipose tissue

## Abstract

**Aims/hypothesis:**

This study aimed to assess the causal relationship between visceral obesity and type 2 diabetes and subsequently to screen visceral adipose tissue (VAT)-specific targets for type 2 diabetes.

**Methods:**

We examined the causal relationship between VAT and type 2 diabetes using bidirectional Mendelian randomisation (MR) followed by multivariable MR. We conducted a transcriptome-wide association study (TWAS) leveraging prediction models and a large-scale type 2 diabetes genome-wide association study (74,124 cases and 824,006 controls) to identify candidate genes in VAT and used summary-data-based MR (SMR) and co-localisation analysis to map causal genes. We performed enrichment and single-cell RNA-seq analyses to determine the cell-specific localisation of the TWAS-identified genes. We also conducted knockdown experiments in 3T3-L1 pre-adipocytes.

**Results:**

MR analyses showed a causal relationship between genetically increased VAT mass and type 2 diabetes (inverse-variance weighted OR 2.48 [95% CI 2.21, 2.79]). Ten VAT-specific candidate genes were associated with type 2 diabetes after Bonferroni correction, including five causal genes supported by SMR and co-localisation: *PABPC4* (1p34.3); *CCNE2* (8q22.1); *HAUS6* (9p22.1); *CWF19L1* (10q24.31); and *CCDC92* (12q24.31). Combined with enrichment analyses, clarifying cell-type specificity with single-cell RNA-seq data indicated that most TWAS-identified candidate genes appear more likely to be associated with adipocytes in VAT. Knockdown experiments suggested that *Pabpc4* likely contributes to regulating differentiation and energy metabolism in 3T3-L1 adipocytes.

**Conclusions/interpretation:**

Our findings provide new insights into the genetic basis and biological processes of the association between VAT accumulation and type 2 diabetes and warrant investigation through further functional studies to validate these VAT-specific candidate genes.

**Graphical Abstract:**

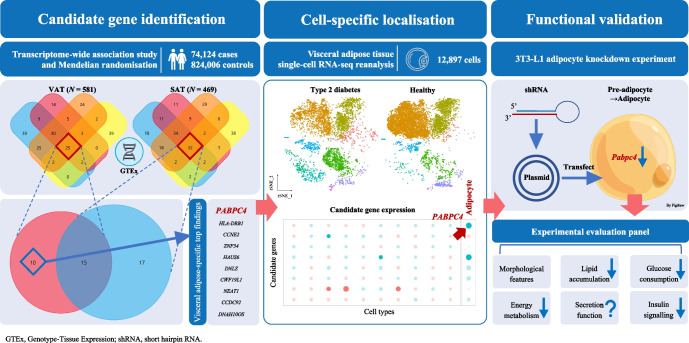

**Supplementary Information:**

The online version of this article (10.1007/s00125-023-05978-5) contains peer-reviewed but unedited supplementary material.

## Introduction


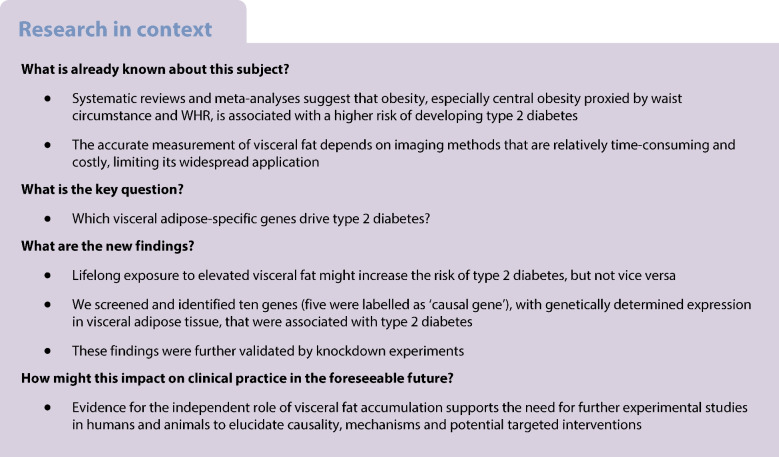


Central adiposity, also known as abdominal adiposity, represents an accumulation of excessive visceral fat. It is usually captured by anthropometric measures such as waist circumference or WHR and has been related to type 2 diabetes independent of BMI. Imaging-based studies have shown that individuals with type 2 diabetes tend to have more visceral fat than BMI-matched individuals without type 2 diabetes [1, 2]. However, these studies were largely limited by small sample sizes and were prone to be influenced by potential environmental and behavioural confounding factors and reverse causation, restricting their ability for causal inference.

It is well known that visceral adipose tissue (VAT) is more harmful than adipose tissue from other locations [3], such as subcutaneous adipose tissue (SAT) and adipose tissue from the extremities. Several studies have focused on the underlying molecular mechanisms and links between visceral fat accumulation and type 2 diabetes, including dysfunctional VAT characterised by adipocyte hypertrophy, the dysregulation of adipocytokines, changes in extracellular matrix (ECM) composition and function, and increased infiltration of inflammatory immune cells [4, 5], each of which may present potential novel therapeutic avenues. Therefore, VAT is a key target organ in which novel molecules and pathways linked to type 2 diabetes pathogenesis could be identified. However, the precise mechanisms require further elucidation.

In the past 10 years, genome-wide association studies (GWAS) have become a powerful tool, not only for revealing the genetic basis and providing the starting point for further studies of potential mechanisms but also for the development of Mendelian randomisation (MR) to make causal inferences in complex diseases. Nevertheless, although more than 200 loci related to type 2 diabetes risk have been identified from the largest GWAS to date [6], it is likely that many loci contributing to type 2 diabetes pathogenesis were missed due to the stringent statistical threshold that is typically used. In addition, the biological interpretation of genetic association results remains a major challenge because most GWAS-identified variants are located in non-coding or intergenic regions, indicating the presence of regulatory mechanisms of gene expression [7, 8]. What is more, these genetic associations carry no information on tissue specificity. Recently, a transcriptome-wide association study (TWAS) was reported to be useful for systematically detecting disease susceptibility genes across various tissues by imputing predicted gene expression into GWAS datasets [9]. TWAS-identified genes are essential to explore disease aetiology, facilitate interpretation of the biological process of GWAS findings and prioritise follow-up functional studies.

In this study, we performed two-sample MR analyses to evaluate the causal effects of genetically increased VAT mass on type 2 diabetes risk. We then utilised the pretrained genetic prediction models established by the three most advanced strategies. We reported results from a large type 2 diabetes TWAS conducted among nearly 900,000 participants of European ancestry to comprehensively identify VAT-specific candidate genes that contribute to type 2 diabetes risk. To validate these in silico findings, we further performed in vitro functional experiments for one gene, *PABPC4*, encoding poly(A) binding protein cytoplasmic 4 (PABPC4).

## Methods

### Study design

**Fig. 1:**
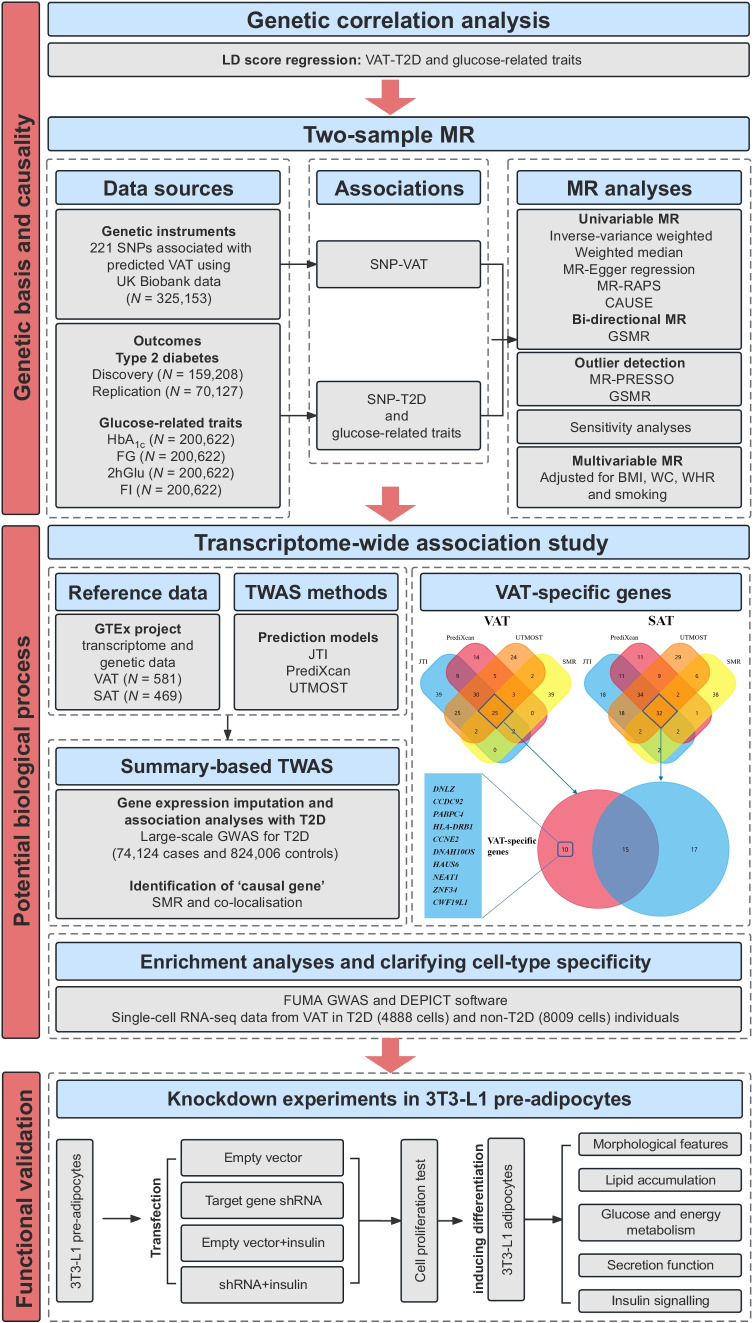
Study design. FI, fasting insulin; T2D, type 2 diabetes; WC, waist circumference

The study flow chart is depicted in Fig. 1. Overall, we utilised a series of MR (inverse-variance weighted [IVW], weighted median, MR-Egger regression, MR-pleiotropy residual sum and outlier [MR-PRESSO], MR-robust adjusted profile score [MR-RAPS], causal analysis using summary effect estimates [CAUSE] and generalised summary-data-based MR [GSMR]) and TWAS, combined with single-cell RNA-seq and enrichment analyses to identify and localise casual genes. Finally, we conducted in vitro experiments to validate the TWAS findings. See ESM Methods for details.

### Univariable and multivariable MR analysis

#### Selection of exposures

The UK Biobank enrolled more than 500,000 participants aged 40–69 years old from the UK between 2006 and 2010. The summary data for predicted VAT mass were acquired from a recent large-scale GWAS (GWAS Catalog ID: GCST008744). We then extracted four variables from the GWAS summary data, including BMI, waist circumference and WHR, from the Genetic Investigation of ANthropometric Traits consortium (GIANT; https://portals.broadinstitute.org/collaboration/giant/index.php/GIANT_consortium_data_files, accessed 19 February 2022) and smoking status from the UK Biobank (downloaded from MR-Base database, https://www.mrbase.org/, accessed on 19 February 2022) to serve as confounding factors to be adjusted in multivariable MR analysis. See ESM Methods for details.

#### Selection of outcomes

We collected the summary data for type 2 diabetes from the DIAbetes Genetics Replication And Meta-analysis (DIAGRAM; http://diagram-consortium.org/downloads.html, accessed 25 February 2022) consortium and 70KforT2D GWAS project (https://kp4cd.org/node/151, accessed 25 February 2022). The glucose-related traits, such as HbA_1c_, fasting glucose (FG), 2h glucose (2hGlu) and fasting insulin, were obtained from the Meta-Analyses of Glucose and Insulin-related traits Consortium (MAGIC; https://magicinvestigators.org/, accessed 27 February 2022). See ESM Methods for details.

### Transcriptome-wide association analysis

We used the recently released data for VAT and SAT from the Genotype-Tissue Expression (GTEx; https://gtexportal.org/home/, accessed 5 March 2022) project (V8), and utilised the pretrained prediction models from Zenodo (https://doi.org/10.5281/zenodo.3842289, accessed 7 March 2022) for further TWAS analysis. See ESM Methods for details.

### In vitro experimental assays for functional validation

We conducted knockdown experiments on mouse 3T3-L1 adipocytes for the genes with the highest priority. Briefly, we used western blot (peroxisome proliferator-activated receptor γ [PPARG], protein kinase AMP-activated catalytic subunit α 1 and 2 [PRKAA1/2], IRS1 and GLUT1), quantitative real-time PCR (qPCR) (*Adipoq*, *Plin1*, *Cidec*, *Lipe*, *Lep* and *Pnpla2*), ELISA (IL-6, TNF-α and monocyte chemoattractant protein-1 [MCP-1] in medium), glucose consumption assays and Oil Red O staining to assess the effects of *Pabpc4* knockdown on the energy and lipid metabolism, secretory functions, glucose utilisation and insulin signalling, respectively, in adipocytes. See ESM Methods for details.

### Statistical analysis

#### LD score regression analysis

We applied linkage disequilibrium (LD) score regression between VAT and type 2 diabetes with GWAS summary statistics using LDSC software version 1.0.1 (https://github.com/bulik/ldsc/, accessed on 1 February 2022). See ESM Methods for details.

#### Univariable and multivariable MR analysis

We performed univariable and bidirectional two-sample MR with seven MR methods, and we took the IVW results as the primary associations while also considering the consistency of the results across other MR methods. See ESM Methods for details.

#### Transcriptome-wide association analysis

We used three different approaches to perform a summary-based TWAS using MetaXcan TWAS pipeline (https://github.com/hakyimlab/MetaXcan, accessed 7 March 2022). See ESM Methods for details.

#### Summary-data-based MR and co-localisation

We performed summary-data-based MR (SMR), heterogeneity in dependent instrument (HEIDI) test, and co-localisation analysis to further screen ‘causal genes’ [*p* (SMR) passing Bonferroni correction, HEIDI *p*>0.05 and posterior probability of a hypothesis (PPH)4 >0.70]. See ESM Methods for details.

#### Bulk RNA-seq and enrichment analysis

We analysed publicly available bulk RNA-seq data from VAT samples of individuals with type 2 diabetes to validate the expression of TWAS-identified genes. We performed functional enrichment analysis to annotate these genes in a biological context using Data-driven Expression Prioritized Integration for Complex Traits tool [10] (DEPICT, https://github.com/perslab/depict, accessed 10 March 2022) and FUMA tool [11] version 1.4.1 (https://github.com/Kyoko-wtnb/FUMA-webapp/). See ESM Methods for details.

#### **S**ingle-cell analysis for the stromal vascular fraction and adipocyte differential gene analysis in VAT

We examined the cell-type-specific expression of the ten candidate genes by using human VAT single-cell RNA-seq data (GEO database accession no. GSE136230). See ESM Methods for details.

#### Analysis software

MR analyses were performed in R (version 4.1.0) [12] with the R packages ‘TwoSampleMR’ (version 0.5.5, https://mrcieu.github.io/TwoSampleMR/) [13], ‘MRPRESSO’ (version 1.0, https://github.com/rondolab/MR-PRESSO) [14], ‘CAUSE’ (version 1.2.0, https://github.com/jean997/cause) [15], ‘gsmr’ (version 1.0.9, https://yanglab.westlake.edu.cn/software/gsmr/) [16] and ‘MVMR’ (version 0.3, https://github.com/WSpiller/MVMR) [17]. TWAS analyses were performed in Python (version 3.9.1) with a Python script in the MetaXcan pipeline. SMR and HEIDI test were performed by the SMR software tool (version 1.3.1; https://yanglab.westlake.edu.cn/software/smr/#Download).

## Results

### Univariable and multivariable MR analyses

**Fig. 2:**
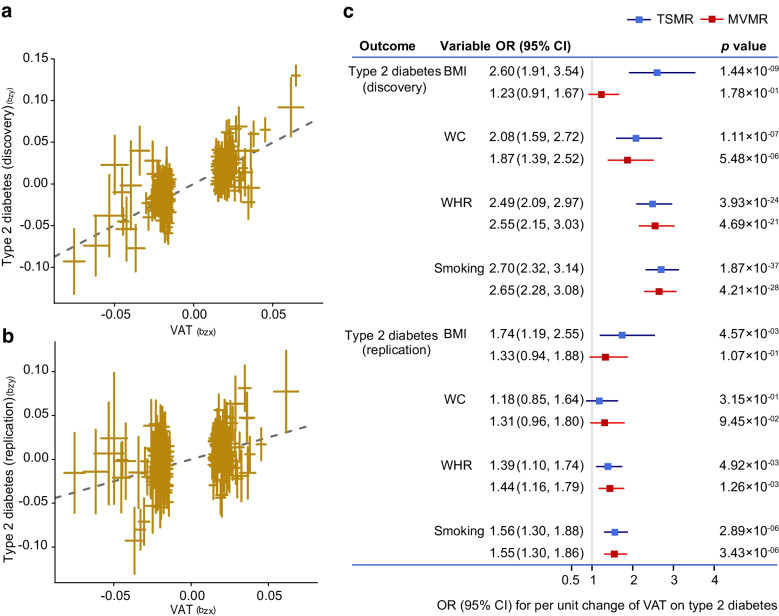
Genetic association between predicted VAT mass and type 2 diabetes risk using GSMR. Scatter plot for discovery analysis (**a**) and replication analysis (**b**), and results of multivariable MR analysis (**c**).b_zx_, correlation between instrumental variables and VAT; b_zy_, correlation between instrumental variables and type 2 diabetes; MVMR, multivariable MR; TSMR, two-sample MR; WC, waist circumference

Participant characteristics and genetic correlations are shown in ESM Results and ESM Tables 1, 2. In total, 221 SNPs were selected as instrumental variables (IVs) for predicted VAT mass based on the IV assumptions in MR analysis (ESM Tables 1, 3 and ESM Fig. 1). Univariable MR showed a causal relationship between the predicted VAT mass and type 2 diabetes risk and HbA_1c_ levels across six MR approaches (VAT and type 2 diabetes with an IVW OR 2.48 [95% CI 2.21, 2.79], and see other results in ESM Results, ESM Table 4 and ESM Figs 2–8). To reduce the possibility of reverse causation, a bidirectional MR was conducted using the GSMR in combination with other MR methods (Table 1, Fig. 2a,b and ESM Table 5). The forward GSMR suggested an adverse effect of the predicted increased VAT on the type 2 diabetes risk (OR 2.70 [95% CI 2.46, 2.96], *p*=3.12×10^−99^ for discovery analysis; OR 1.65 [95% CI 1.46, 1.87], *p*=1.05×10^−15^ for replication analysis). The reverse GSMR showed a significant association between genetically determined type 2 diabetes and a higher VAT mass; however, the ORs were very close to the null value (0.99 [95% CI 0.98, 1.00]). Notably, the causal relationships from other reverse MR (using two-sample MR analysis) were no longer significant (ESM Table 5). There was little evidence to support an association between genetically increased glucose-related traits and VAT mass (ESM Table 4). Sensitivity analysis showed evidence of heterogeneity but without horizontal pleiotropy between VAT and type 2 diabetes (see ESM Results ‘Sensitivity analysis’ and ESM Table 6).

**Table 1:** Bidirectional GSMR analysis for predicted VAT on the risk of type 2 diabetes

Outcome Type 2 diabetes	Forward		Reverse		Pleiotropic outliers (no. of SNPs)	
	OR (95% CI)	*p* value	OR (95% CI)	*p* value	Forward	Reverse
Discovery cohort	2.70 (2.46, 2.96)	3.12×10^−99^	0.99 (0.98, 1.00)	6.09×10^−3^	7^a^	8^b^
Replication cohort	1.65 (1.46, 1.87)	1.05×10^−15^	0.99 (0.98, 1.00)	1.34×10^−2^	6^c^	2^d^

^a^rs10423928, rs111363146, rs2744973, rs329124, rs429358, rs7649970 and rs8074454 were identified as outliers

^b^rs10189235, rs10768984, rs11642015, rs17036160, rs429358, rs4565329, rs62007299 and rs703976 were identified as outliers

^c^rs10896012, rs2102278, rs329124, rs72663503, rs7649970 and rs8074454 were identified as outliers

^d^rs3768321 and rs71304101 were identified as outliers

Taking into account the results of the univariable MR analysis, multivariable MR was performed to further determine the independent effects of VAT adjusted for potential confounders. We found that the strong positive associations between VAT and type 2 diabetes persisted using the two multivariable MR methods (except for the association adjusted for BMI, which was attenuated). The multivariable-adjusted ORs (95% CIs) were 2.60 (1.91, 3.54) adjusted for BMI, 2.08 (1.59, 2.72) adjusted for waist circumference, 2.49 (2.09, 2.97) adjusted for WHR and 2.70 (2.32, 3.14) adjusted for smoking status (Fig. 2c). Although the relationship between the predicted VAT and type 2 diabetes was attenuated after adjustment for waist circumference in replication analysis, it still showed the same effect direction and overlapped CIs as the univariable results (Fig. 2c).

### Transcriptome-wide association analysis

Using joint-tissue imputation (JTI), PrediXcan and modified unified test for molecular signatures (UTMOST), three separate prediction models were built and detailed information on the prediction performance for each gene is shown in ESM Tables 7–12. The JTI framework, PrediXcan and UTMOST prioritised 132 genes (Bonferroni-corrected *p*<4.04×10^−6^), 88 genes (*p*<6.14×10^−6^) and 116 genes (*p*<5.34×10^−6^), respectively. The Manhattan plots using the three TWAS methods are shown in ESM Fig. 9. As a next step, in our gene-prioritisation strategy, the SMR prioritised 42 candidate genes. Finally, 25 genes were identified by all methods.

To compare the expression of genes contributing to type 2 diabetes risk with VAT, the same analyses were repeated to identify candidate genes expressed in SAT using GTEx data. Finally, 32 genes were identified by all methods. There were ten VAT-related genes (five novel type 2 diabetes-associated genes) showing no significant gene-level TWAS associations in SAT-related analysis and thus they represent independent hits (Table 2). At the 1p34.3 locus, genetically elevated expression of *PABPC4* was associated with decreased type 2 diabetes risk. Besides, genetically elevated expression of *CCNE2* on 8q22.1, *HAUS6* on 9p22.1, *CWF19L1* on 10q24.31, *CCDC92* on 12q24.31 and *DNAH10OS* on 12q24.31 showed protective effects on type 2 diabetes risk. All these associations were further filtered by co-localisation between the gene expression and type 2 diabetes associations (PPH4 >0.70) and the SMR HEIDI test (*p*>0.05). Four other genes identified in the TWAS failed to be confirmed by co-localisation analysis or the SMR HEIDI test. The detailed TWAS and SMR results are shown in ESM Tables 7–14.

**Table 2:** TWAS identified ten VAT-specific genes that associated with type 2 diabetes

Gene	Chr	JTI		PrediXcan		UTMOST		SMR		Co-localisation					Causal gene
		*z* score	*p* value	*z* score	*p* value	*z* score	*p* value	*p* value	HEIDI	PPH0	PPH1	PPH2	PPH3	PPH4	
*PABPC4*	1p34.3	−6.86	6.98×10^−12^	−6.88	6.18×10^−12^	−8.24	1.72×10^−16^	1.18×10^–7^	0.409	<0.001	<0.001	<0.001	0.103	0.897	Yes
*HLA-DRB1*	6p21.32	−6.66	2.80×10^−11^	−5.91	3.43×10^–9^	−7.09	1.39×10^−12^	2.57×10^–6^	0.005	<0.001	<0.001	<0.001	1	<0.001	No
*CCNE2*^a^	8q22.1	−6.42	1.41×10^−10^	−6.11	9.72×10^−10^	−6.21	5.31×10^−10^	1.85×10^–6^	0.461	<0.001	<0.001	<0.001	0.284	0.716	Yes
*ZNF34*^a^	8q24.3	5.34	9.51×10^–8^	6.10	1.06×10^–9^	5.44	5.46×10^–8^	1.35×10^–6^	0.129	<0.001	<0.001	<0.001	1	<0.001	No
*HAUS6*^a^	9p22.1	−5.60	2.10×10^–8^	−5.75	8.72×10^–9^	−5.13	2.85×10^–7^	8.56×10^–7^	0.673	<0.001	<0.001	<0.001	0.265	0.735	Yes
*DNLZ*	9q34.3	7.59	3.21×10^−14^	7.10	1.27×10^−12^	7.60	2.94×10^−14^	1.12×10^–6^	0.003	<0.001	<0.001	<0.001	1	<0.001	No
*CWF19L1*	10q24.31	−5.04	4.70×10^–7^	−4.92	8.78×10^–7^	−4.93	8.37×10^–7^	7.92×10^–6^	0.100	<0.001	<0.001	<0.001	0.065	0.928	Yes
*NEAT1*^a^	11q13.1	5.34	9.35×10^–8^	6.40	1.60×10^−10^	6.23	4.53×10^−10^	2.34×10^–7^	0.006	<0.001	<0.001	<0.001	0.991	0.009	No
*CCDC92*	12q24.31	−6.95	3.71×10^−12^	−7.15	8.79×10^−13^	−6.33	2.46×10^−10^	1.31×10^–6^	0.139	<0.001	<0.001	<0.001	0.198	0.802	Yes
*DNAH10OS*^a^	12q24.31	−5.98	2.27×10^–9^	−5.61	2.05×10^–8^	−5.70	1.21×10^–8^	7.08×10^–7^	0.043	<0.001	<0.001	<0.001	0.077	0.923	No

^a^These diabetes-related genes have not been reported by previous GWAS

### Bulk RNA-seq and enrichment analysis

**Fig. 3:**
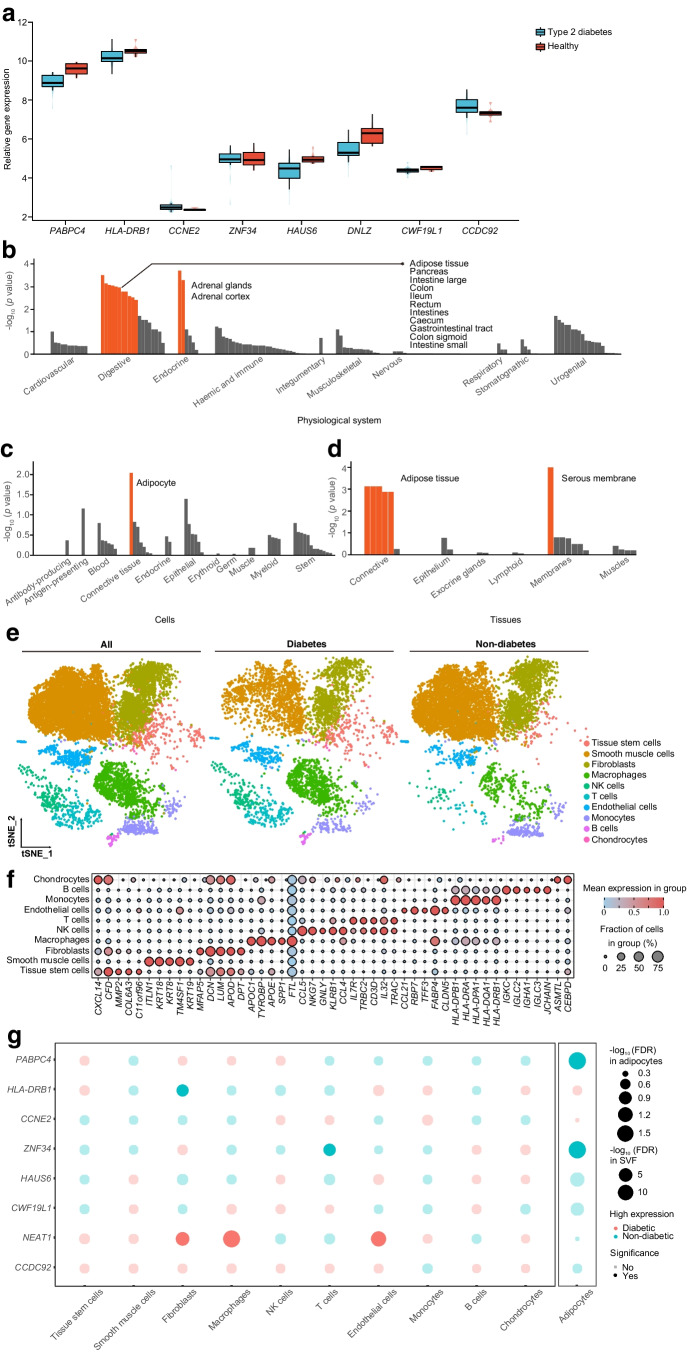
Cellular localisation of TWAS-identified genes. (**a**) Relative expression (count) of TWAS-identified genes in bulk RNA-seq data GSE71416 (this dataset did not include long non-coding RNA: *NEAT1* and *DNAH10OS*). The boxes represent the interquartile range (IQR) containing the middle 50% of the data, from Q1 to Q3. The horizontal line indicates the median value of the gene expression data. The whiskers extend from the box to the smallest and largest observations within 1.5 × IQR, representing the spread of the data. (**b**–**g**) DEPICT enrichment analysis and single-cell RNA-seq analysis for cell-type specificity: tissue and cell-type enrichment analyses including physiological systems (**b**), cells (**c**) and tissues (**d**); the orange columns indicate where *p* values were corrected by the false discovery rate correction (FDR<0.05); (**e**) identified cell populations in the VAT; (**f**) top differentially expressed genes; and (**g**) dot plot of differential expression for TWAS-identified genes between type 2 diabetes and controls (*DNLZ* and *DNAH10OS* were filtered due to their low expression levels). FDR, false discover rate; NK, natural killer; tSNE, t-distributed stochastic neighbor embedding

According to the analysis of bulk RNA-seq data, three significantly differentially expressed TWAS-identified genes were observed when comparing individuals with type 2 diabetes and non-diabetic individuals with morbid obesity: *PABPC4* (*p*=0.004); *HAUS6* (*p*=0.011); and *DNLZ* (*p*=0.013). Moreover, *HLA-DRB1* (*p*=0.086) and *CWF19L1* (*p*=0.100) reached the margin of significance (Fig. 3a).

Adipose tissue and adipocytes showed the strongest enrichment using the DEPICT tool (Fig. 3b–d and ESM Table 15). A pathway analysis for JTI-identified genes using FUMA detected several significantly associated Gene Ontology gene sets. The top five sets were MHC protein complex assembly (*p*=6.26×10^−7^), activating transcription factor 6 (ATF6)-mediated unfolded protein response (*p*=3.72×10^−6^), negative regulation of insulin secretion (*p*=6.64×10^−6^), negative regulation of signalling (*p*=7.26×10^−6^) and cell cycle process (*p*=7.32×10^−6^). The results for all enriched pathways with a false discovery rate of <0.05 are presented in ESM Table 16.

### Single-cell analysis for the stromal vascular fraction and adipocytes differential gene analysis in VAT

Enrichment analysis suggested that adipocytes might be associated with type 2 diabetes risk. It is important to note that adipose tissue is a complex and highly active organ that is composed of two main cell populations (adipocytes and the stromal vascular fraction [SVF; immune cells, pre-adipocytes, endothelial cells, etc.]). Therefore, single-cell RNA-seq data of SVF in VAT were re-analysed. After filtering, 12,897 SVF cells (4888 cells from individuals with type 2 diabetes and 8009 cells from individuals without diabetes) were obtained. Annotation of the clusters using recommended marker genes resulted in the following ten cell types: tissue stem cells; smooth muscle cells; fibroblasts; macrophages; natural killer (NK) cells; T cells; endothelial cells; monocytes; B cells; and chondrocytes (Fig. 3e), showing different expression patterns (Fig. 3f). The cell distribution in individuals with type 2 diabetes was clearly distinct from that in individuals without diabetes (Fig. 3e). Notably, most TWAS-identified genes did not show differential expression between the two groups, except for a higher expression of *NEAT1* in fibroblasts, macrophages and endothelial cells in individuals with type 2 diabetes and a higher expression of *HLA-DRB1* and *ZNF34* in fibroblasts and T cells in individuals without diabetes (Fig. 3g). In addition, *PABPC4* and *ZNF34* differed in their expression patterns in adipocytes (Fig. 3g). In general, among these findings, *PABPC4* exhibited a good agreement at different omics levels, including genetics, bulk RNA-seq (Fig. 3a; two additional GEO datasets are shown in ESM Fig. 10) and single-cell RNA-seq analysis.

### In vitro experimental assays for functional validation

**Fig. 4:**
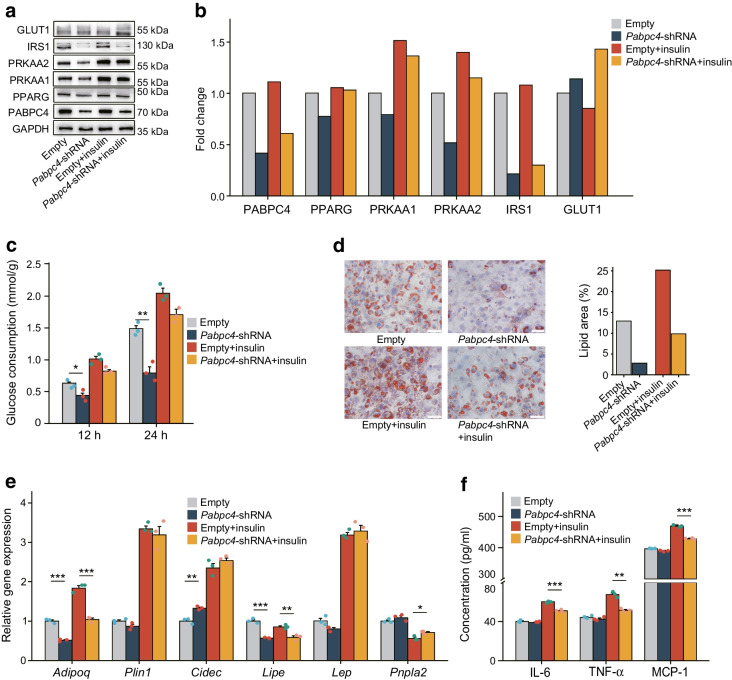
*Pabpc4* knockdown experiments in mouse 3T3-L1 adipocytes. (**a**) Western blot for energy metabolism-related proteins. (**b**) Protein expression fold change. (**c**) Glucose consumption at 12 h and 24 h. (**d**) Oil Red O staining. (**e**) Expression of lipid metabolism-related genes (calculated by ${2}^{{-\Delta \Delta \mathrm{C}}_{\mathrm{t}}}$: ΔC_t_ refers to the difference in threshold cycle values between the target gene and a reference gene, and ΔΔC_t_ represents the difference in ΔC_t_ values between different experimental groups). (**f**) Assessment of the secretory function of maturing adipocytes, showing concentrations in the supernatant fractions of adipocyte cultures. Scale bar, 50 μm. *p<0.05, **p<0.01, ***p<0.001

Based on the above analysis of the results, the expression of *PABPC4* in adipocytes was speculated to be related to type 2 diabetes risk. This finding was strengthened further by a series of knockdown experiments in mouse 3T3-L1 adipocytes. The experimental materials, reagents and primer sequences are shown in ESM Tables 17–18. *Pabpc4* knockdown was confirmed by conducting both western blot and qPCR analysis, both showing a significant decrease compared with the control group transfected with empty vector. Knockdown of *Pabpc4* inhibited the expression of PPARG (tested before the induction of the differentiation), PRKAA1/2 and IRS1 under basal conditions, representing the regulation of cell differentiation, energy metabolism and insulin signalling, respectively. Only GLUT1 was upregulated especially in the insulin-stimulated group (Fig. 4a). Further, similar changes in protein expression were noted in response to *Pabpc4* knockdown under insulin conditions (Fig. 4a,b). Glucose consumption experiments showed that knockdown of *Pabpc4* inhibited the glucose utilisation of 3T3-L1 adipocytes (Fig. 4c). Given the effect of *Pabpc4* knockdown on glucose uptake, insulin signalling experiments with p-Akt assessment were conducted, showing a significant downregulation in Akt signalling (ESM Fig. 11). Oil Red O staining indicated that *Pabpc4* might be associated with the lipid storage capacity of adipocytes (Fig. 4d). Measurement of lipid metabolism-related genes showed that *Adipoq* and *Lipe* were downregulated while *Cidec* was upregulated (Fig. 4e). The supernatant fractions of adipocyte cell cultures were assayed for IL-6, TNF-α and MCP-1, showing a consistent downregulation under insulin conditions (Fig. 4f).

## Discussion

In this study, we provided evidence that VAT mass was genetically correlated with type 2 diabetes and HbA_1c_ using whole-genome LDSC. Two-sample and bidirectional MR analyses suggested that genetically determined VAT mass was causally related to an increased risk of type 2 diabetes and a higher level of HbA_1c_ but not vice versa. Furthermore, utilising the reference data for VAT and SAT from the GTEx project, TWAS analyses identified ten VAT-specific genes related to type 2 diabetes risk. Of these, five putative target genes, including *PABPC4* on 1p34.3, *CCNE2* on 8q22.1, *HAUS6* on 9p22.1, *CWF19L1* on 10q24.31 and *CCDC92* on 12q24.31, were labelled ‘causal genes’ according to the SMR and co-localisation analyses. Knockdown experiments in 3T3-L1 adipocytes indicated that *Pabpc4* may have a role in regulating the differentiation and energy metabolism.

The genetic relationship between obesity-related traits (e.g. BMI, waist circumference and WHR) and type 2 diabetes has already been established [18, 19] but this relationship has not been established for VAT. A population-based twin study found a significantly higher genetic correlation between insulin resistance and central fat (*r* = 0.41) than that for total fat (0.24) [20]. These findings illustrated that central fat is not only a predictor of insulin resistance but also shares genetic influence with insulin resistance, which is a major factor in obesity-linked type 2 diabetes. We found significant genetic correlations of VAT with type 2 diabetes and HbA_1c_, but not FG, 2hGlu or fasting insulin, suggesting that VAT accumulation might be associated with persistent dysglycaemia. Furthermore, most observational studies have specifically investigated the association between imaging-measured VAT and type 2 diabetes risk with a limited sample size. A recent study, using dual-energy x-ray absorptiometry (DXA) to accurately measure VAT in more than 4000 participants, reported a strong association between VAT mass and type 2 diabetes risk (OR [95% CI] 8.55 [4.48, 16.3] for female participants and 2.01 [1.56, 2.58] for male participants). VAT was a stronger risk factor in the female participants while the higher overall prevalence of type 2 diabetes in the male participants could be ascribed to their larger average depot of VAT [21]. These associations were verified by MR analyses, suggesting a causal role of the predicted VAT on type 2 diabetes [21].

Nevertheless, the sample size of that study was relatively small (9517 type 2 diabetes cases), and the MR estimations were not adjusted for potential confounders such as BMI. In comparison, our MR analyses leveraged a larger sample size (26,676 type 2 diabetes cases in discovery analysis and 12,931 cases in replication analysis) to make causal inference and obtained an independent effect of VAT that was independent from BMI, waist circumference, WHR and smoking status. These analyses indicated that targeting body fat distribution, especially VAT, might be a promising strategy for type 2 diabetes. However, the specific mechanisms involved in the pathogenesis of type 2 diabetes associated with VAT remain to be elucidated. Thus, the candidate genes identified by TWAS could provide a new direction for unravelling the shared aetiologies between VAT and type 2 diabetes since genetic regulatory variation is the major determinant of target gene expression and subsequently alters the levels of proteins [22, 23].

Usually, there are two possibilities for the relationship between gene expression alterations and disease status: one is that the altered gene expression leads to disease conditions (potential targets); and the other is that the disease itself results in aberrant gene expression (biomarkers). Using TWAS analyses, several candidate genes identified in both VAT and SAT have been studied more extensively. For example, IRS1 is a substrate of insulin receptor tyrosine kinase, which plays an important role in insulin-stimulated signal transduction pathway. Kilpeläinen et al identified a locus near *IRS1* that was associated with increased body fat but it was a robustly protective locus for cardiometabolic risk including type 2 diabetes and coronary artery disease [24]. Juxtaposed with another zinc finger protein 1 (JAZF1) is a transcriptional coregulator associated with pathophysiological processes in type 2 diabetes, as confirmed by animal studies. Overexpression of JAZF1 inhibits glucose production by the liver [25]. It also acts as an important regulator of endoplasmic reticulum stress, preventing p53-mediated metabolic stress in beta cells [26]. *WFS1* mutation leads to the Wolfram syndrome, of which the most common clinical phenotypes are early onset diabetes and neurological symptoms. *Wfs1*-knockout rats show a strikingly different diabetic phenotype compared with previous animal models [27].

However, the aforementioned genes cannot reflect the unique contribution made by VAT to type 2 diabetes. We identified ten VAT-specific genes that were related to type 2 diabetes. These included eight protein-coding genes, three of which were verified in a bulk RNA-seq dataset. For several of our identified genes, there is already evidence from published studies supporting their potential role in the pathogenesis of type 2 diabetes. For instance, *PABPC4* is localised primarily to the cytoplasm and is necessary for regulating the stability of labile mRNA. Furthermore, it has been identified as a putative functional gene for type 2 diabetes from the SMR analysis in the Consortium for the Architecture of Gene Expression (CAGE, *N*=2765 in peripheral blood) [28]. It was also reported that *PABPC4* is necessary for the regulation of fat and glucose levels, especially when high-fat diets are consumed. Thus, it may form part of the important link between obesity and type 2 diabetes [29]. Consistently, our in vitro functional assays verified that the knockdown of *Pabpc4* decreases glucose utilisation in 3T3-L1 adipocytes, and this process is less likely to be mediated by glucose transporters (GLUT1 was upregulated). Interestingly, it has been reported that glucose entrance through GLUT1 with activation of the hexosamine pathway may decrease the insulin-mediated glucose transport through GLUT4, leading to insulin resistance [30]. In contrast, AMP-activated protein kinase (AMPK), a key cellular sensor of energy balance that regulates energy metabolism [31], may be a molecular signalling pathway downstream of PABPC4. We observed that the expression levels of PRKAA1/2 and p-Akt were downregulated in the shRNA groups. Therefore, we speculate that the effect of *Pabpc4* knockdown on glucose consumption may be achieved through the AMPK and Akt signalling pathway.

Moreover, the decreased *Pparg* expression suggested that *Pabpc4* may be involved in pre-adipocyte differentiation, which has been shown to be related to adipocyte insulin resistance [32]. Examination of lipid metabolism-related genes provided some clues to explain how *Pabpc4* knockdown affects adipocyte lipid storage capacity. For example, *Adipoq* gene promotes glucose uptake and fatty acid oxidation in adipocytes and regulates adipocyte energy metabolism and insulin sensitivity [33]. *Lipe*, encoding kinase-sensitive lipase (HSL), is a key enzyme in triacylglycerol catabolism in adipocytes [34]. *Cidec* has been proven to be involved in the formation and maintenance of lipid droplets in adipocytes. Knockdown of *Cidec* in well-differentiated adipocytes enhances lipolysis, increases the number of mitochondria and increases the expression of genes associated with brown adipocyte identity [35].

Other TWAS-identified causal genes, such as *CWF19L1*, are likely to be involved in cell cycle regulation and are well conserved in several species. However, there have been no experimental studies that link the expression of *CWF19L1* with type 2 diabetes. Previous research has shown that common variants of the *ERLIN1*–*CHUK*-CWF19L1 gene cluster act in fatty liver and metabolic diseases [36]. *CCDC92* encodes a coiled-coil domain protein that can interact with proteins at the centriole–ciliary interface. In a bivariate GWAS, a novel signal at rs825476 near *CCDC92*, which is also an expression quantitative trait locus (eQTL) for this gene, was identified as a shared locus between type 2 diabetes and CHD [37]. The effects of two other causal genes, *CCNE2* and *HAUS6*, on type 2 diabetes have not been validated in functional studies and will need to be investigated in future research.

### Strength and limitations

To the best of our knowledge, this study is the first to systematically identify VAT-specific candidate genes that are associated with type 2 diabetes risk using TWAS analyses. TWAS-identified genes are more appropriately interpreted as prioritised genes at relative loci, meaning that these genes do not imply causality. Thus, we used different approaches, such as TWAS, SMR and co-localisation, to confirm and complement each other, making the results more reliable. Moreover, we re-analysed and combined the single-cell RNA-seq data to determine the cell-type specificity for these candidate genes.

Notably, it should be emphasised that there are also three major limitations in our study. First, the relatively small sample size for GTEx reference data of VAT limits the precision and power to detect associations less of moderate strength. VAT is a heterogeneous tissue containing many cell types (organ-specific and migratory) with relative proportions that can vary according to the harvesting location and environmental factors. Second, TWAS only focuses on genes with significant *cis*-heritability (*cis*-eQTLs, within a certain distance from gene body). However, the expression of some genes can be regulated by *trans*-regulatory elements in the human genome; thus, the importance of *trans*-eQTLs in transcriptional regulation should not be overlooked. Third, the reference data we used in TWAS were mainly of European ancestry due to the lack of large-scale eQTL data from other ancestry groups, diseases, medical conditions, sex, etc., limiting the generalisability of the findings to other ethnic groups.

### Conclusions

In summary, our findings provide substantial new information to improve our understanding of the genetics and aetiology of type 2 diabetes and lay a foundation for further functional studies of these identified target genes to thoroughly explore the in-depth mechanisms.

### Supplementary Information

Below is the link to the electronic supplementary material.

Supplementary file1 (PDF 5196 KB)

Supplementary file2 (XLSX 9234 KB)

## Data Availability

All data generated or analysed during this study are included in this main text.

## Abbreviations

2hGlu: 2 h glucose
AMPK: AMP-activated protein kinase
CAUSE: Causal Analysis Using Summary Effect Estimates
DIAGRAM: DIAbetes Genetics Replication And Meta-analysis
ECM: Extracellular matrix
eQTL: Expression quantitative trait locus
FG: Fasting glucose
GIANT: Genetic Investigation of ANthropometric Traits consortium
GSMR: Generalised summary-data-based MR
GTEx: Genotype-Tissue Expression
GWAS: Genome-wide association study
HEIDI: Heterogeneity in dependent instrument
IV: Instrumental variable
IVW: Inverse-variance weighted
JTI: Joint-tissue imputation
LD: Linkage disequilibrium
MAGIC: Meta-Analyses of Glucose and Insulin-related traits Consortium
MCP-1: Monocyte chemoattractant protein-1
MR: Mendelian randomisation
MR-PRESSO: MR-pleiotropy residual sum and outlier
MR-RAPS: MR-robust adjusted profile score
PABPC4: Poly(A) binding protein cytoplasmic 4
PPH: Posterior probability of a hypothesis
qPCR: Quantitative real-time PCR
SAT: Subcutaneous adipose tissue
SMR: Summary-data-based MR
SVF: Stromal vascular fraction
TWAS: Transcriptome-wide association study
UTMOST: Unified test for molecular signatures
VAT: Visceral adipose tissue
