# Supplementary material for: Transcriptome-wide association study-derived genes as potential visceral adipose tissue-specific targets for type 2 diabetes
Source: Diabetologia. 2023 Aug 4;66(11):2087–100. doi: 10.1007/s00125-023-05978-5 (PMC10542736; doi:10.1007/s00125-023-05978-5)
Supplement: Supplementary file 1 — Supplementary file1 (PDF 5196 KB) [file 125_2023_5978_MOESM1_ESM.pdf]

ESM Methods

Study design

The study flow chart is depicted in Fig. 1. First, we selected genetic variants as instrumental variables (IVs) ($p < 5\times10^{-8}$) for VAT and retrieved the complete summary data from the GWASs for type 2 diabetes (one for discovery and the other for replication analysis) and glucose-related traits. Second, we performed univariable and bidirectional two-sample MR with seven MR methods, including inverse-variance weighted (IVW), weighted median, MR-Egger regression, MR-Pleiotropy Residual Sum and Outlier (MR-PRESSO), MR-Robust adjusted profile score (MR-RAPS), Causal Analysis Using Summary Effect Estimates (CAUSE), and Generalized Summary-data-based Mendelian Randomisation (GSMR). Third, we conducted a series of sensitivity analyses and multivariable MR adjusted for body mass index (BMI), waist circumstance (WC), waist-hip ratio (WHR), and smoking status. Fourth, we did a TWAS to identify transcribed VAT-specific candidate genes for which expression is related to type 2 diabetes risk. Fifth, we identified specific cell types in VAT with specific expression of the candidate genes using DEPICT software and publicly available single-cell transcriptomic data. Finally, we conducted knockdown experiments in 3T3-L1 preadipocytes to validate the TWAS findings.

Univariable and multivariable MR analyses

Selection of exposures

The UK Biobank enrolled more than 500,000 participants aged 40-69 years old from the United Kingdom between 2006 and 2010, and it contains the in-depth genetic and health information of these participants. This study aimed to identify phenotypic and health-related information by following up with participants over time. All participants gave written informed consent for data collection, analysis, and record linkage.

The summary data of predicted VAT mass in this study were based on a recent large-scale GWAS, which was constructed of two subcohorts. One subcohort was called the VAT-training dataset. In this subcohort the VAT was measured by dual energy X-ray absorptiometry (DXA, GE Healthcare Lunar iDXA scanner) in 4198 individuals of white British ancestry and used to create prediction models. The other subcohort was called the VAT-application dataset. This subcohort included 325,153 individuals, and the VAT was calculated according to the prediction models [coefficient of determination = 0.76 (0.74 to 0.78)] [1]. GWAS summary data for the predicted VAT are available at https://www.ebi.ac.uk/gwas/downloads/summary-statistics (Study Accession ID: GCST008744).

Similarly, we extracted four parameters of the GWAS summary data to serve as confounding factors to be adjusted in multivariable MR analysis. BMI was obtained from a meta-analysis of GWASs including 681,275 participants [2]. WC and WHR were obtained from the Genetic Investigation of ANthropometric Traits consortium (GIANT, https://portals.broadinstitute.org/collaboration/giant/index.php/GIANT_consortium_data_files) including 336,639 and 210,082 participants, respectively. The data for smoking status were obtained from the UK Biobank including 462,434 participants (available data extracted from the MR-Base database) [3].

Among the SNPs available in each GWAS summary dataset, we selected SNPs robustly associated with exposures as IVs ($p < 5\times10^{-8}$, IV Assumption 1, ESM Fig. 1). To minimize the linkage disequilibrium (LD) effects that lead to nonrandomized allele allocation, a relatively stringent condition (LD threshold of $r^2 < 0.001$ and distance located 10000 kb apart from each other) was set to ensure that the selected IVs were conditionally independent of each other. The *F*-statistic represents the strength of the relationship between the IVs and exposures. Generally, an *F*-statistic > 10 provides evidence against the possibility

of bias produced by weak IVs [4].

Selection of outcomes

We collected the summary data of the discovery type 2 diabetes cohort obtained from the DIAbetes Genetics Replication And Meta-analysis (DIAGRAM, http://diagram-consortium.org/downloads.html) consortium (26,676 cases and 132,532 controls with a mean age of 57.4 years old) [5]. For the replication analysis, we collected the summary data of another type 2 diabetes cohort from the 70KforT2D project (70KforT2D, https://t2d.hugeamp.org/), including 12,931 cases and 57,196 controls [6]. In addition, we also included glucose-related traits, such as glycated hemoglobin (HbA1c), fasting glucose (FG), 2h-glucose (2hGlu), and fasting insulin (FI), into our MR analysis. These data were obtained from the Meta-Analyses of Glucose and Insulin-related traits Consortium (MAGIC, https://magicinvestigators.org/), which is a meta-analysis of 91 studies with 200,622 participants [7].

The participants had an identical genetic background (European ancestry), and to our knowledge, there was no sample overlap between the exposure and outcome GWASs.

Transcriptome-wide association analysis

We used the recently released data of the Genotype-Tissue Expression (GTEx, https://gtexportal.org/home/) project (V8), which includes RNA sequencing data and whole-genome sequencing (WGS) data of VAT (omentum, N = 581) and SAT (N = 469). Detailed information on RNA sequencing experiments, WGS, and quality control of these data have been described elsewhere [8]. The training methods of gene-expression models can be found in previous studies [9-11]. We utilized the pre-trained prediction models from Zenodo (https://doi.org/10.5281/zenodo.3842289) for further

transcriptome-wide association analyses. We collected the summary data of type 2 diabetes for TWAS from the DIAGRAM consortium, which was the largest meta-analysis of GWASs, including 74,124 cases and 824,006 controls of European ancestry [12].

In vitro experimental assays for functional validation

Mouse 3T3-L1 preadipocytes were purchased from the Cell Bank of the Chinese Academy of Sciences. A complete list of the other materials and reagents used is provided (ESM Table 17). The pLKO.1-puro (addgene, 10878) was used to knock down gene *Pabpc4* with validation using quantitative RT-PCR according to previous studies [13]. The culture and differentiation of 3T3-L1 cells have been described elsewhere [14], and we made small modifications according to the actual conditions. Briefly, the cells were cultured and maintained in high-glucose DMEM containing 10% FBS and 1% penicillin/streptomycin in a 5% CO_2 environment. The cells were allowed to grow for 2 days after confluency. Then, they were induced by incubating the cells with DMEM containing 1.0 μg/mL insulin, 1.0 μM DEX, 0.5 mM IBMX, and 10% FBS for 2 days and DMEM containing 1.0 μg/mL insulin and 10% FBS for another 3 days. The medium was then changed every 2 days until completion of the differentiation process, and adipocytes were maintained in DMEM medium supplemented with 10% FBS, after which time the mature 3T3-L1 adipocytes were used for experiments. The mature 3T3-L1 adipocytes were divided into four groups: empty (control), *Pabpc4*-shRNA, empty+insulin, and *Pabpc4*-shRNA+insulin group, with three replicates for each group. Of these, the insulin treatment was done for last two groups with an hour and a concentration of 100 nM.

Oil-red-o staining was performed to detect the lipid content and distribution in adipocytes as previously described [15]. The cultured medium of 3T3-L1 adipocyte cultures was collected, and the glucose content

in the supernatant was measured by the GOD-POD glucose kit. The absorbance at 505 nm was measured by a spectrophotometer, and the absorbance of the standard tube and each tube to be measured was read by zeroing the blank tube. Glucose consumption was measured in mmol/protein amounts.

Quantitative RT-PCR was performed previously described [16]. Notably, *Pparg* expression will be examined before the induction of the differentiation (on preadipocytes). The primer sequences used in this study are provided (ESM Table 18). The methods for western blot and ELISA have been described elsewhere [17].

Statistical analysis

LD score regression analysis

To evaluate whether type 2 diabetes and glucose-related traits are genetically correlated with VAT, we applied LD score regression with GWAS summary statistics using LDSC software (all default parameters see the website https://github.com/bulik/ldsc/) [18, 19]. We used precomputed LD scores based on the European ancestry samples from the 1000 Genomes Project [20] using the GWAS summary statistics of HapMap3 SNPs. Only autosomal SNPs with MAF > 5% were included in the analysis, and SNPs in the MHC region were not included due to the long-range LD. Moreover, we calculated the heritability of type 2 diabetes and glucose-related traits.

Univariable and multivariable MR analyses

As shown in ESM Fig. 1, we estimated the causal effect of the predicted VAT on type 2 diabetes and glucose-related traits using a classic MR model [21, 22]. Ideally, a valid IV should satisfy the following three assumptions (ESM Fig. 1): (1) must be truly associated with VAT (in this study, defined as the $p <$

5×10^{-8}); (2) not associated with confounders of VAT and type 2 diabetes and glucose-related traits; and (3) should only be associated with type 2 diabetes and glucose-related traits through VAT.

To evaluate the causal effects of VAT on the risk of type 2 diabetes and glucose-related traits by combining multiple SNPs, we conducted a univariable and bidirectional two-sample MR with seven MR methods, including IVW [23], weighted median [24], MR-Egger regression [25], MR-PRESSO [26], MR-RAPS [27], CAUSE [28], and GSMR [29]. IVW is a conventional method that is used to obtain an MR estimate by performing a meta-analysis of each Wald ratio for multiple SNPs; the largest statistical power among all MR methods occurs when the selected variants are all valid IVs. The weighted median estimator determines the median effect of SNPs, providing a valid estimation even when including 50% of the invalid IVs. MR-Egger regression, with a relaxed criterion, allows the presence of horizontal pleiotropy across SNPs. Moreover, it also requires the InSIDE (Instrument Strength Independent of Direct Effect) assumption to be satisfied [25]. Notably, the MR-Egger regression has less power and provides wider confidence intervals than the IVW. MR-PRESSO is a unified framework evaluating horizontal pleiotropy in a standard MR model to detect outlier SNPs and provide a corrected MR result. In addition, we used the MR-RAPS to reduce weak instrument bias. The CAUSE models correlated and uncorrelated the horizontal pleiotropy, avoiding possible false-positives when using other MR methods. Compared with other MR methods based on summary statistics, GSMR provides greater statistical power because GSMR uses genome-wide data to account for the sampling variance between SNPs and exposure-outcome estimations.

To test whether IV assumptions were violated, we evaluated the heterogeneity of the results using Cochran's Q-test [30] and detected the potential presence of horizontal pleiotropy using the MR-Egger intercept test, MR-PRESSO global test, and HEIDI test in GSMR. We performed leave-one-out analyses

by eliminating SNPs separately and recomputing the effect. We also assessed the possible directional pleiotropy by observing the symmetry characteristics of funnel plots similar to those used to assess publication bias in the meta-analysis. Once heterogeneity or horizontal pleiotropy was noted, we recomputed the MR results after removing the outlier SNPs identified by the MR-PRESSO and HEIDI tests in GSMR.

Obesity-related traits, such as BMI, WC, and WHR, are highly correlated with VAT and have been reported to be related to type 2 diabetes [31-33]. Thus, we further used MVMR analysis to estimate the direct causal effects of VAT on the risk of type 2 diabetes independent of the effects of BMI, WC, and WHR. Additionally, smoking status is associated with increased insulin resistance and central fat accumulation [34, 35]. Therefore, we added this potential confounder to the multivariable-adjusted models.

We took the IVW results as the primary associations while also considering the consistency of the results across other MR methods. In this study, we defined the evidence for a potential causal effect of predicted VAT on the type 2 diabetes risk when the following criteria were met: (1) at least six MR methods results (including IVW) had a Bonferroni corrected p-value $< 8.33\times10^{-3}$ (0.05/6); (2) at least one of the multivariable MR results (calculated by two-sample MR or MVMR) showed a similar effect size to the IVW (p-value < 0.05); (3) other MR methods provided effect sizes similar to those of IVW and MVMR; and (4) the Egger intercept test provided a p-value > 0.05 and the funnel plots showed a symmetrical distribution of SNP effects.

Transcriptome-wide association analysis

We performed a summary-based TWAS using three different approaches, including the recently

developed joint-tissue imputation (JTI) method [9], the PrediXcan [10], and the modified unified test for molecular signatures (UTMOST) [9, 11] to establish genetic prediction models of normal VAT gene expression using the MetaXcan TWAS pipeline. Overall, the JTI borrows information on each tissue-tissue pair (or cell type) and estimates the gene expression profile similarity and the epigenetic similarity (chromatin accessibility evaluated by the DNase I hypersensitivity sites in the promoter region) to improve the prediction quality. The PrediXcan uses the elastic net with fivefold cross-validation to determine the optimal hyperparameter. Similarly, UTMOST borrows information across tissues and significantly increases the prediction accuracy by using a sparse group-LASSO method. Zhou et al. [9] developed the modified UTMOST framework using uniform hyperparameters across different folds to make the hyperparameters directly comparable. It has been confirmed in external datasets that the modified UTMOST provides an approximately unbiased estimate of prediction performance. For these three prediction models, genes with good prediction quality from fivefold cross-validation ($r > 0.1$ and $p < 0.05$) were defined as imputable genes and were used for downstream analyses. Then, we constructed a transcriptome model from VAT samples and SNP covariance matrices built from 1000 Genome reference samples. Finally, we investigated the associations between the predicted gene expression in VAT and type 2 diabetes risk using GWAS summary statistics generated from the DIAGRAM consortium (64,124 cases and 824,006 controls). We applied Bonferroni corrections for multiple comparisons, taking into account the total number of tested genes across different methods. We considered the associations to be significant when $p_{(TWAS)} < 4.04\times10^{-6}$ (0.05/12377) in JTI, $p_{(TWAS)} < 6.14\times10^{-6}$ (0.05/8140) in PrediXcan, and $p_{(TWAS)} < 5.34\times10^{-6}$ (0.05/9367) in modified UTMOST.

Summary-data-based Mendelian randomisation and colocalisation

We further screened the TWAS results using the Summary-data-based Mendelian randomisation (SMR) followed by the heterogeneity in dependent instrument (HEIDI) test [36] and colocalisation analysis [37]. The SMR was used to test for the potential causal effect of the expression level of a gene (multiple variants in the cis-eQTL region as the IVs) on a complex trait of interest using summary GWAS data and expression quantitative trait loci (eQTLs) studies, which can be used to prioritize genes for follow-up functional studies. Colocalisation analysis was performed to evaluate whether the observed eQTLs in VAT and type 2 diabetes GWAS associations were consistent with a shared SNP in a given region (1 Mb on both sides of this gene body). We considered the associations in SMR to be significant when the Bonferroni corrected $p_{(SMR)} < 8.55\times10^{-6}$ (0.05/5849) and HEIDI $p > 0.05$, and a posterior probability of a hypothesis (PPH4, gene expressions, and type 2 diabetes are associated and share a single causal SNP) of 70% or higher was considered evidence of colocalisation.

To identify the VAT-specific genes that contribute to type 2 diabetes risk, we performed the same analyses using SAT data from the GTEx project, including TWAS, SMR, and colocalisation.

Bulk RNA-seq analysis and enrichment

To observe the actual expression of TWAS-identified genes, we analyzed publicly available bulk RNA-seq data of VAT samples from fourteen individuals with type 2 diabetes and six without type 2 diabetes (GEO database with accession number: GSE71416) [38].

For the tissue and cell-type enrichment analysis, we used the Data-driven Expression Prioritized Integration for Complex Traits (DEPICT) tool [39] to identify tissues and cell types where genes from associated loci are highly expressed. We further performed functional enrichment analysis for the genes associated with type 2 diabetes risk. We utilized the GENE2FUNC function in the FUMA tool to annotate

the TWAS-identified genes in a biological context [40].

Single-cell analysis for the SVF and adipocytes differential gene analysis in VAT

We examined the cell type-specific expression of the ten candidate genes by using human VAT single-cell RNA-seq data profiled from Vijay et al. [41]. First, we divided this scRNA-seq cohort, consisting of four males and ten females, into two groups: the type 2 diabetes (five individuals) and the nondiabetic (nine individuals) groups. Then, we performed the transformation on the raw single-cell RNA-seq data (GEO database with accession number: GSE136230) using Seurat (version 2.3.4) [42]. The key parameters in the quality control step were consistent with those in the original study [41]. We performed principal component analysis (PCA) using the top 23 dimensions. Clusters were annotated using overlapping known marker genes among the cluster-specific genes. Finally, we performed differential expression analysis to determine whether these candidate genes were differentially expressed in a particular cell type between the two groups.

MR analyses were performed in R (version 4.1.0) with the R packages "TwoSampleMR" [3], "MRPRESSO" [26], "CAUSE" [28], "gsmr" [29], and "MVMR" [43]. TWAS analyses were performed in Python (version 3.9.1) with a Python script in the MetaXcan pipeline.

ESM Results

Participant characteristics and instruments for MR analyses

The characteristics of the participants from UK Biobank, GIANT, and consortia of type 2 diabetes and glucose-related traits (DIAGRAM, 70KforT2D, and MAGIC) are shown in ESM Table 1. In total, 221

SNPs were selected as IVs (ESM Table 3) for predicted VAT mass with an *F*-statistic of 901.13, reflecting a strong instrument strength. In addition, there were 490, 225, 38, and 35 SNPs for BMI, WC, WHR, and smoking status, respectively, that were used as IVs to perform the multivariable MR analysis.

Genetic correlations of predicted VAT with type 2 diabetes and glucose-related traits

According to the LD score regression results, the predicted VAT was genetically correlated with type 2 diabetes ($r_g = 0.575$; 95% CI 0.508, 0.642; $p = 7.85\times10^{-66}$ for DIAGRAM, and $r_g = 0.331$; 95% CI 0.262, 0.400; $p = 9.21\times10^{-21}$ for 70KforT2D) and one of the glucose-related traits (HbA1c: $r_g = 0.195$; 95% CI 0.140, 0.250; $p = 1.23\times10^{-12}$). No significant genetic correlations were observed between the predicted VAT and other glucose-related traits, including FG, 2hGlu, and FI.

Two-sample MR results

Univariable and bidirectional MR

The results of univariable MR analysis for the effect of increased VAT on the risk of type 2 diabetes and glucose-related traits are shown in ESM Table 4. For discovery analysis, genetically increased VAT was associated with a higher risk for type 2 diabetes (IVW: OR = 2.48; 95% CI 2.21, 2.79; $p = 9.28\times10^{-54}$). Other MR methods, such as weighted median, MR-Egger, and MR-RAPS, demonstrated good consistency with the IVW (all $p < 8.33\times10^{-3}$). MR-PRESSO detected and removed outlier variants, providing a corrected estimation (OR = 2.51; 95% CI 2.24, 2.81; $p = 3.83\times10^{-38}$). This finding was also supported by the CAUSE (OR = 1.95; 95% CI 1.77, 2.16; $p = 6.50\times10^{-10}$), after accounting for correlated and uncorrelated horizontal pleiotropy and controlling for possible false-positives. A similar result was observed across all MR methods for replication analysis, suggesting a causal relationship between

genetically determined VAT and type 2 diabetes risk except for the MR-Egger regression (OR = 1.50; 95% CI 0.93, 2.42; $p = 9.80\times10^{-2}$). In addition, the genetically increased VAT mass was associated with HbA1c levels (IVW: OR = 1.04; 95% CI 1.02, 1.05; $p = 1.02\times10^{-7}$), which was also confirmed by other MR methods. There was little evidence to support an association between genetically increased VAT mass and other glucose-related factors, such as FG, 2hGlu, and FI.

Sensitivity analyses

A series of sensitivity analyses were used to evaluate the heterogeneity and potential horizontal pleiotropy (ESM Table 6). Cochran's Q-test showed evidence ($p < 0.05$) for the presence of heterogeneity across all MR estimations. The MR-Egger intercept tests indicated the absence of unbalanced horizontal pleiotropy ($p > 0.05$) across all MR estimations except for FI [$p_{(intercept)} = 0.047$]. The leave-one-out tests found that no instances when sequentially dropping one a single SNP out led to dramatic changes in the overall estimations (ESM Fig. 2-7). The funnel plots showed a symmetrical distribution of variant effects for type 2 diabetes and HbA1c, indicating an absence of directional pleiotropy (ESM Fig. 2-4). The scatter plots generated by CAUSE are shown in ESM Fig. 8.

References

1. Karlsson T, Rask-Andersen M, Pan G, et al (2019) Contribution of genetics to visceral adiposity and its relation to cardiovascular and metabolic disease. Nat Med 25(9):1390-1395. http://doi.org/10.1038/s41591-019-0563-7
2. Shungin D, Winkler TW, Croteau-Chonka DC, et al (2015) New genetic loci link adipose and insulin biology to body fat distribution. Nature 518(7538):187-196. http://doi.org/10.1038/nature14132
3. Hemani G, Zheng J, Elsworth B, et al (2018) The MR-Base platform supports systematic causal inference across the human phenome. eLife 7. http://doi.org/10.7554/eLife.34408
4. Burgess S, Thompson SG (2011) Avoiding bias from weak instruments in Mendelian randomization studies. Int J Epidemiol 40(3):755-764. http://doi.org/10.1093/ije/dyr036
5. Scott RA, Scott LJ, Mägi R, et al (2017) An Expanded Genome-Wide Association Study of Type 2

Diabetes in Europeans. Diabetes 66(11):2888-2902. http://doi.org/10.2337/db16-1253
6. Bonàs-Guarch S, Guindo-Martínez M, Miguel-Escalada I, et al (2018) Re-analysis of public genetic data reveals a rare X-chromosomal variant associated with type 2 diabetes. Nature Communications 9(1):321. http://doi.org/10.1038/s41467-017-02380-9
7. Chen J, Spracklen CN, Marenne G, et al (2021) The trans-ancestral genomic architecture of glycemic traits. Nat Genet 53(6):840-860. http://doi.org/10.1038/s41588-021-00852-9
8. Battle A, Brown CD, Engelhardt BE, Montgomery SB (2017) Genetic effects on gene expression across human tissues. Nature 550(7675):204-213. http://doi.org/10.1038/nature24277
9. Zhou D, Jiang Y, Zhong X, Cox NJ, Liu C, Gamazon ER (2020) A unified framework for joint-tissue transcriptome-wide association and Mendelian randomization analysis. Nat Genet 52(11):1239-1246. http://doi.org/10.1038/s41588-020-0706-2
10. Gamazon ER, Wheeler HE, Shah KP, et al (2015) A gene-based association method for mapping traits using reference transcriptome data. Nat Genet 47(9):1091-1098. http://doi.org/10.1038/ng.3367
11. Hu Y, Li M, Lu Q, et al (2019) A statistical framework for cross-tissue transcriptome-wide association analysis. Nat Genet 51(3):568-576. http://doi.org/10.1038/s41588-019-0345-7
12. Mahajan A, Taliun D, Thurner M, et al (2018) Fine-mapping type 2 diabetes loci to single-variant resolution using high-density imputation and islet-specific epigenome maps. Nat Genet 50(11):1505-1513. http://doi.org/10.1038/s41588-018-0241-6
13. Pietrobono S, Anichini G, Sala C, et al (2020) ST3GAL1 is a target of the SOX2-GLI1 transcriptional complex and promotes melanoma metastasis through AXL. Nat Commun 11(1):5865. http://doi.org/10.1038/s41467-020-19575-2
14. Zhang Y, Ye M, Chen LJ, Li M, Tang Z, Wang C (2015) Role of the ubiquitin-proteasome system and autophagy in regulation of insulin sensitivity in serum-starved 3T3-L1 adipocytes. Endocr J 62(8):673-686. http://doi.org/10.1507/endocrj.EJ15-0030
15. Mehlem A, Hagberg CE, Muhl L, Eriksson U, Falkevall A (2013) Imaging of neutral lipids by oil red O for analyzing the metabolic status in health and disease. Nat Protoc 8(6):1149-1154. http://doi.org/10.1038/nprot.2013.055
16. Uzureau P, Uzureau S, Lecordier L, et al (2013) Mechanism of Trypanosoma brucei gambiense resistance to human serum. Nature 501(7467):430-434. http://doi.org/10.1038/nature12516
17. Gassen NC, Hartmann J, Zschocke J, et al (2014) Association of FKBP51 with priming of autophagy pathways and mediation of antidepressant treatment response: evidence in cells, mice, and humans. PLoS Med 11(11):e1001755. http://doi.org/10.1371/journal.pmed.1001755
18. Bulik-Sullivan BK, Loh PR, Finucane HK, et al (2015) LD Score regression distinguishes confounding from polygenicity in genome-wide association studies. Nat Genet 47(3):291-295. http://doi.org/10.1038/ng.3211
19. Bulik-Sullivan B, Finucane HK, Anttila V, et al (2015) An atlas of genetic correlations across human diseases and traits. Nat Genet 47(11):1236-1241. http://doi.org/10.1038/ng.3406
20. Abecasis GR, Auton A, Brooks LD, et al (2012) An integrated map of genetic variation from 1,092 human genomes. Nature 491(7422):56-65. http://doi.org/10.1038/nature11632
21. Zheng J, Baird D, Borges MC, et al (2017) Recent Developments in Mendelian Randomization Studies. Curr Epidemiol Rep 4(4):330-345. http://doi.org/10.1007/s40471-017-0128-6
22. Smith GD, Ebrahim S (2003) 'Mendelian randomization': can genetic epidemiology contribute to understanding environmental determinants of disease? Int J Epidemiol 32(1).
23. Johnson T, Uk S (2012) Efficient calculation for multi-SNP genetic risk scores.

24. Bowden J, Davey Smith G, Haycock PC, Burgess S (2016) Consistent Estimation in Mendelian Randomization with Some Invalid Instruments Using a Weighted Median Estimator. Genet Epidemiol 40(4):304-314. http://doi.org/10.1002/gepi.21965
25. Bowden J, Davey Smith G, Burgess S (2015) Mendelian randomization with invalid instruments: effect estimation and bias detection through Egger regression. Int J Epidemiol 44(2):512-525. http://doi.org/10.1093/ije/dyv080
26. Verbanck M, Chen C-Y, Neale B, Do R (2018) Detection of widespread horizontal pleiotropy in causal relationships inferred from Mendelian randomization between complex traits and diseases. Nat Genet 50(5):693-698. http://doi.org/10.1038/s41588-018-0099-7
27. Zhao Q, Wang J, Bowden J, Small D (2018) Statistical inference in two-sample summary-data Mendelian randomization using robust adjusted profile score. Annals of Statistics 48. http://doi.org/10.1214/19-AOS1866
28. Morrison J, Knoblauch N, Marcus JH, Stephens M, He X (2020) Mendelian randomization accounting for correlated and uncorrelated pleiotropic effects using genome-wide summary statistics. Nat Genet 52(7):740-747. http://doi.org/10.1038/s41588-020-0631-4
29. Zhu Z, Zheng Z, Zhang F, et al (2018) Causal associations between risk factors and common diseases inferred from GWAS summary data. Nature Communications 9(1):224. http://doi.org/10.1038/s41467-017-02317-2
30. Burgess S, Bowden J, Fall T, Ingelsson E, Thompson SG (2017) Sensitivity Analyses for Robust Causal Inference from Mendelian Randomization Analyses with Multiple Genetic Variants. Epidemiology (Cambridge, Mass) 28(1):30-42.
31. Teufel F, Seiglie JA, Geldsetzer P, et al (2021) Body-mass index and diabetes risk in 57 low-income and middle-income countries: a cross-sectional study of nationally representative, individual-level data in 685 616 adults. Lancet 398(10296):238-248. http://doi.org/10.1016/s0140-6736(21)00844-8
32. Feller S, Boeing H, Pischon T (2010) Body mass index, waist circumference, and the risk of type 2 diabetes mellitus: implications for routine clinical practice. Dtsch Arztebl Int 107(26):470-476. http://doi.org/10.3238/arztebl.2010.0470
33. Vazquez G, Duval S, Jacobs DR, Jr., Silventoinen K (2007) Comparison of body mass index, waist circumference, and waist/hip ratio in predicting incident diabetes: a meta-analysis. Epidemiol Rev 29:115-128. http://doi.org/10.1093/epirev/mxm008
34. Eliasson B (2003) Cigarette smoking and diabetes. Prog Cardiovasc Dis 45(5):405-413. http://doi.org/10.1053/pcad.2003.00103
35. Chiolero A, Faeh D, Paccaud F, Cornuz J (2008) Consequences of smoking for body weight, body fat distribution, and insulin resistance. Am J Clin Nutr 87(4):801-809. http://doi.org/10.1093/ajcn/87.4.801
36. Zhu Z, Zhang F, Hu H, et al (2016) Integration of summary data from GWAS and eQTL studies predicts complex trait gene targets. Nat Genet 48(5):481-487. http://doi.org/10.1038/ng.3538
37. Giambartolomei C, Vukcevic D, Schadt EE, et al (2014) Bayesian test for colocalisation between pairs of genetic association studies using summary statistics. PLoS Genet 10(5):e1004383. http://doi.org/10.1371/journal.pgen.1004383
38. Doumatey AP, Xu H, Huang H, et al (2015) Global Gene Expression Profiling in Omental Adipose Tissue of Morbidly Obese Diabetic African Americans. J Endocrinol Metab 5(3):199-210. http://doi.org/10.14740/jem286w
39. Pers TH, Karjalainen JM, Chan Y, et al (2015) Biological interpretation of genome-wide association

studies using predicted gene functions. Nat Commun 6:5890. http://doi.org/10.1038/ncomms6890
40. Watanabe K, Taskesen E, van Bochoven A, Posthuma D (2017) Functional mapping and annotation of genetic associations with FUMA. Nature Communications 8(1):1826. http://doi.org/10.1038/s41467-017-01261-5
41. Vijay J, Gauthier MF, Biswell RL, et al (2020) Single-cell analysis of human adipose tissue identifies depot and disease specific cell types. Nat Metab 2(1):97-109. http://doi.org/10.1038/s42255-019-0152-6
42. Butler A, Hoffman P, Smibert P, Papalexi E, Satija R (2018) Integrating single-cell transcriptomic data across different conditions, technologies, and species. Nat Biotechnol 36(5):411-420. http://doi.org/10.1038/nbt.4096
43. Sanderson E, Davey Smith G, Windmeijer F, Bowden J (2018) An examination of multivariable Mendelian randomization in the single-sample and two-sample summary data settings. Int J Epidemiol 48(3):713-727. http://doi.org/10.1093/ije/dyy262

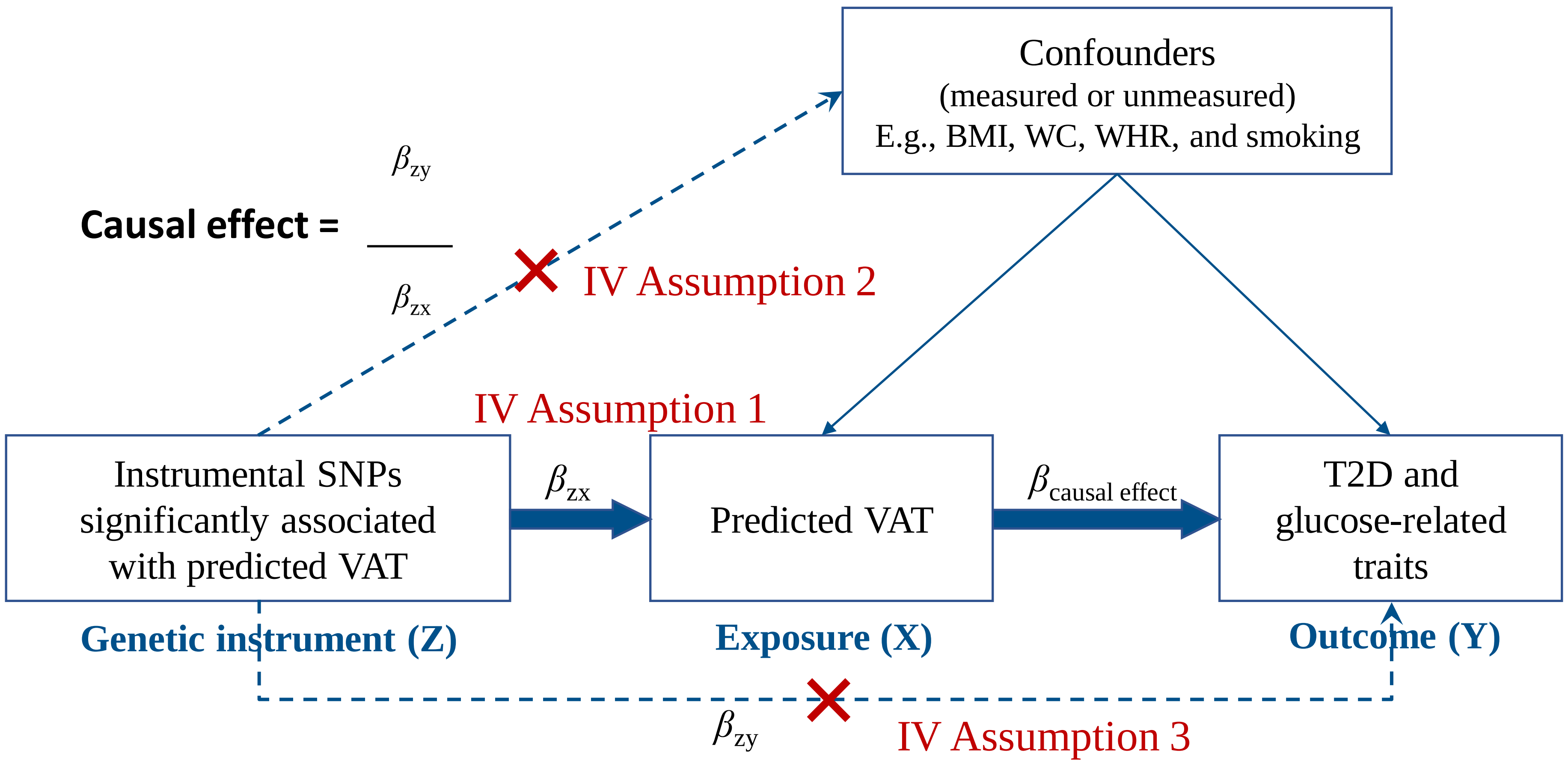

ESM Fig 1. Instrumental variable (IV) assumptions of Mendelian randomisation.

BMI indicates body mass index; WC, waist circumference; WHR, waist-to-hip ratio; SNP, single nucleotide polymorphism; VAT, visceral adipose tissue; T2D, type 2 diabetes.

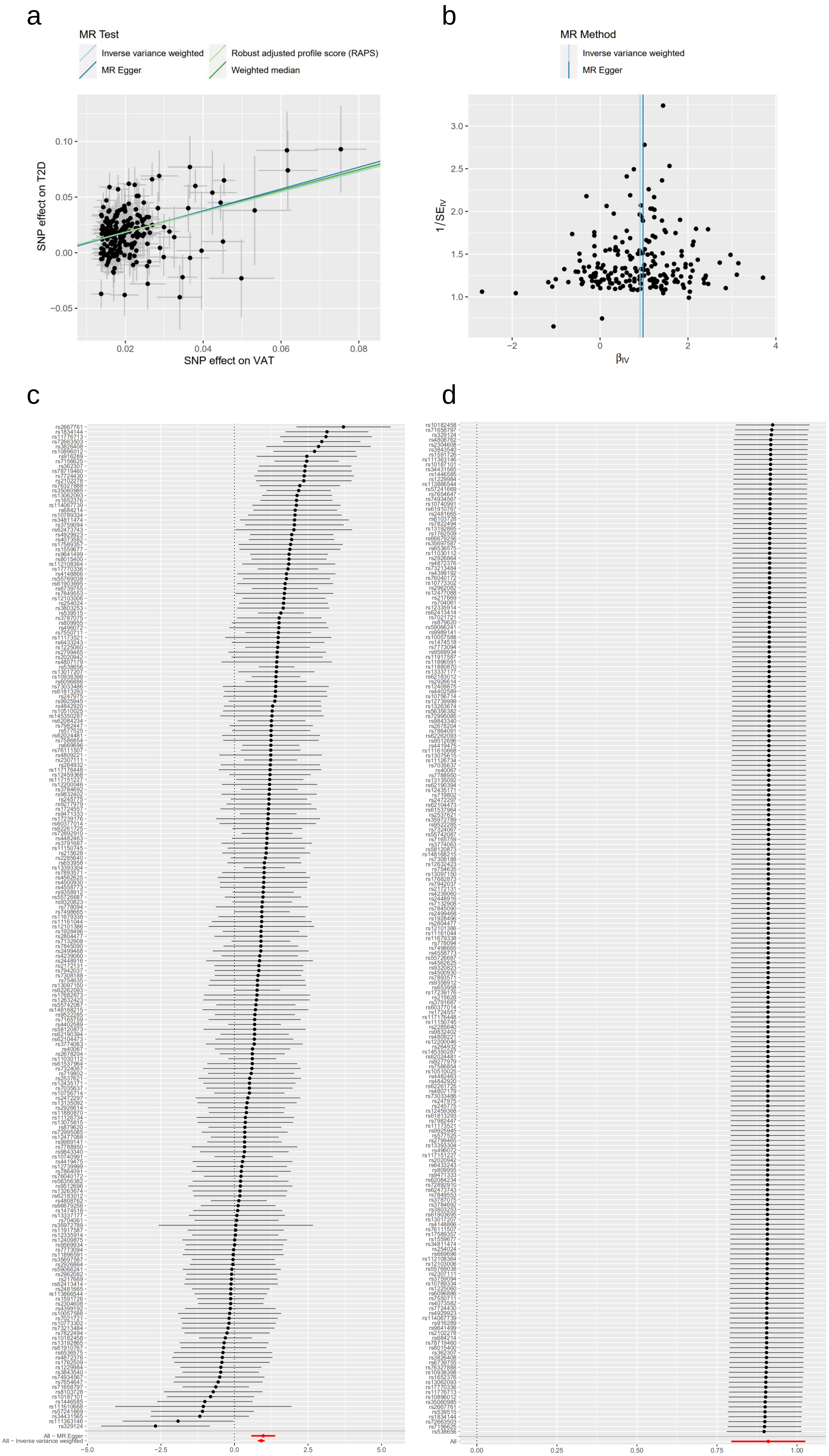

ESM Fig. 2. Scatter plot (a), funnel plot (b), forest plot (c), and leave-one-out test (d) for VAT on T2D (discovery analysis).

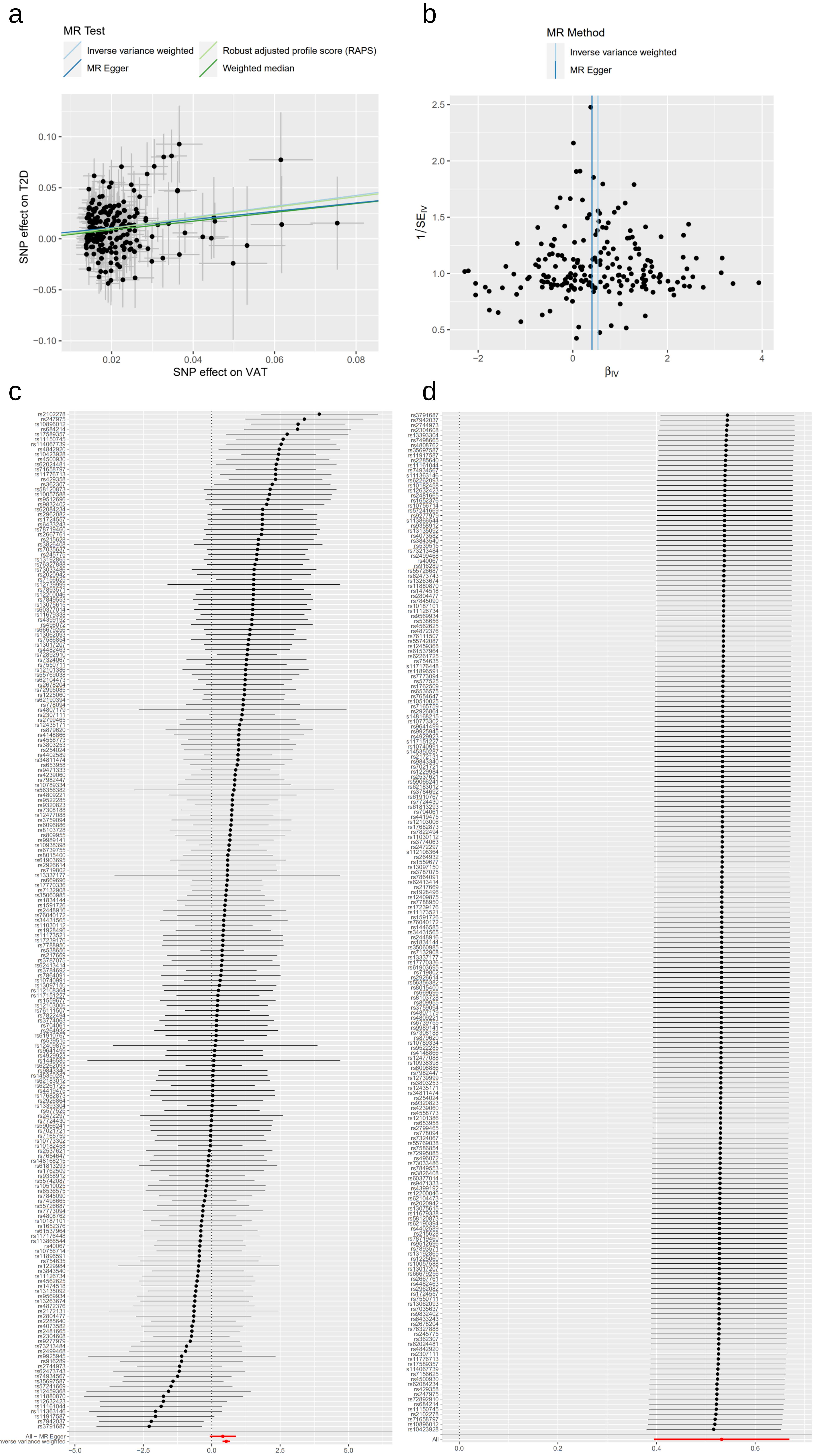

ESM Fig. 3. Scatter plot (a), funnel plot (b), forest plot (c), and leave-one-out test (d) for VAT on T2D (replication analysis).

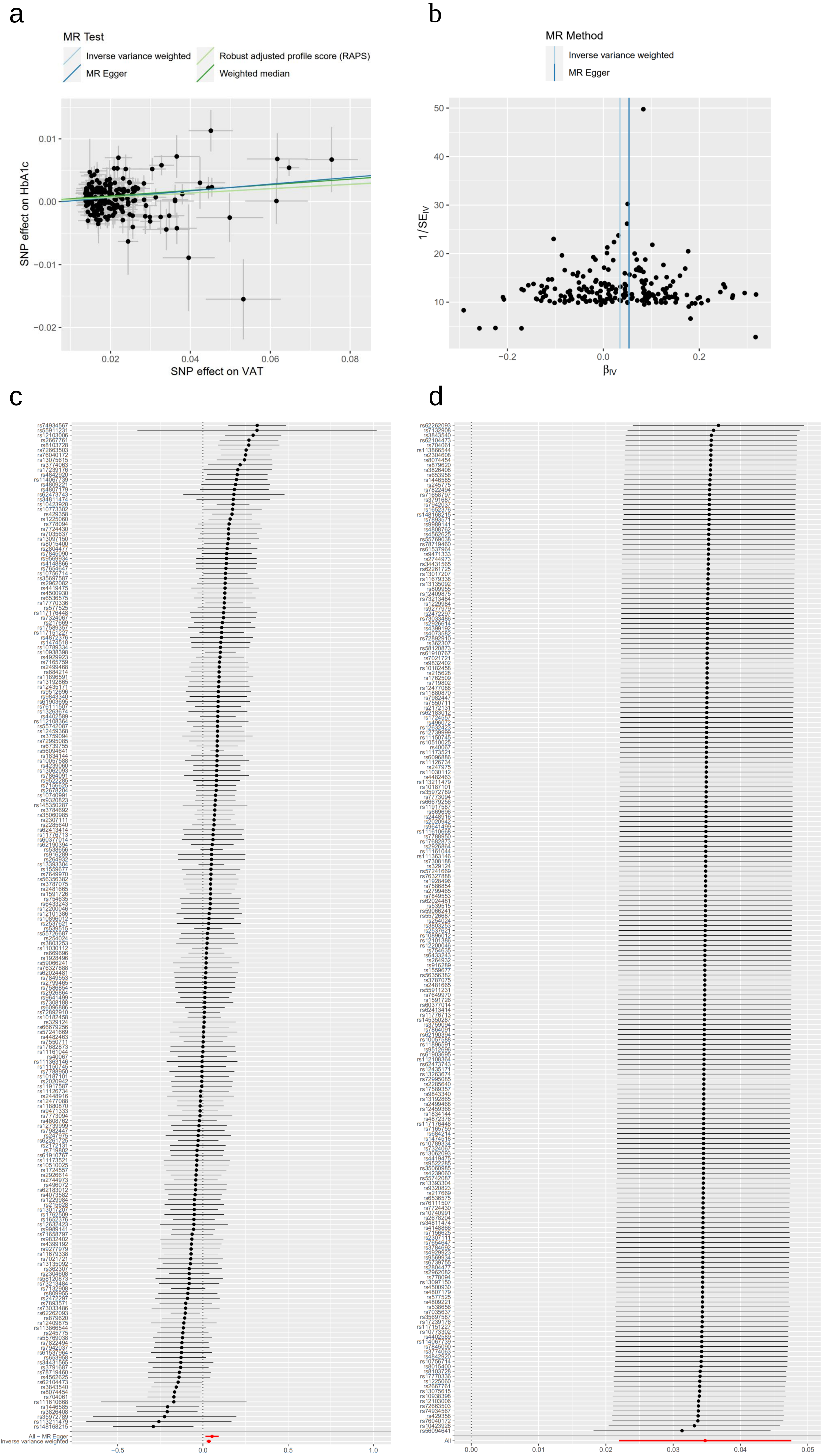

ESM Fig. 4. Scatter plot (a), funnel plot (b), forest plot (c), and leave-one-out test (d) for VAT on HbA1c.

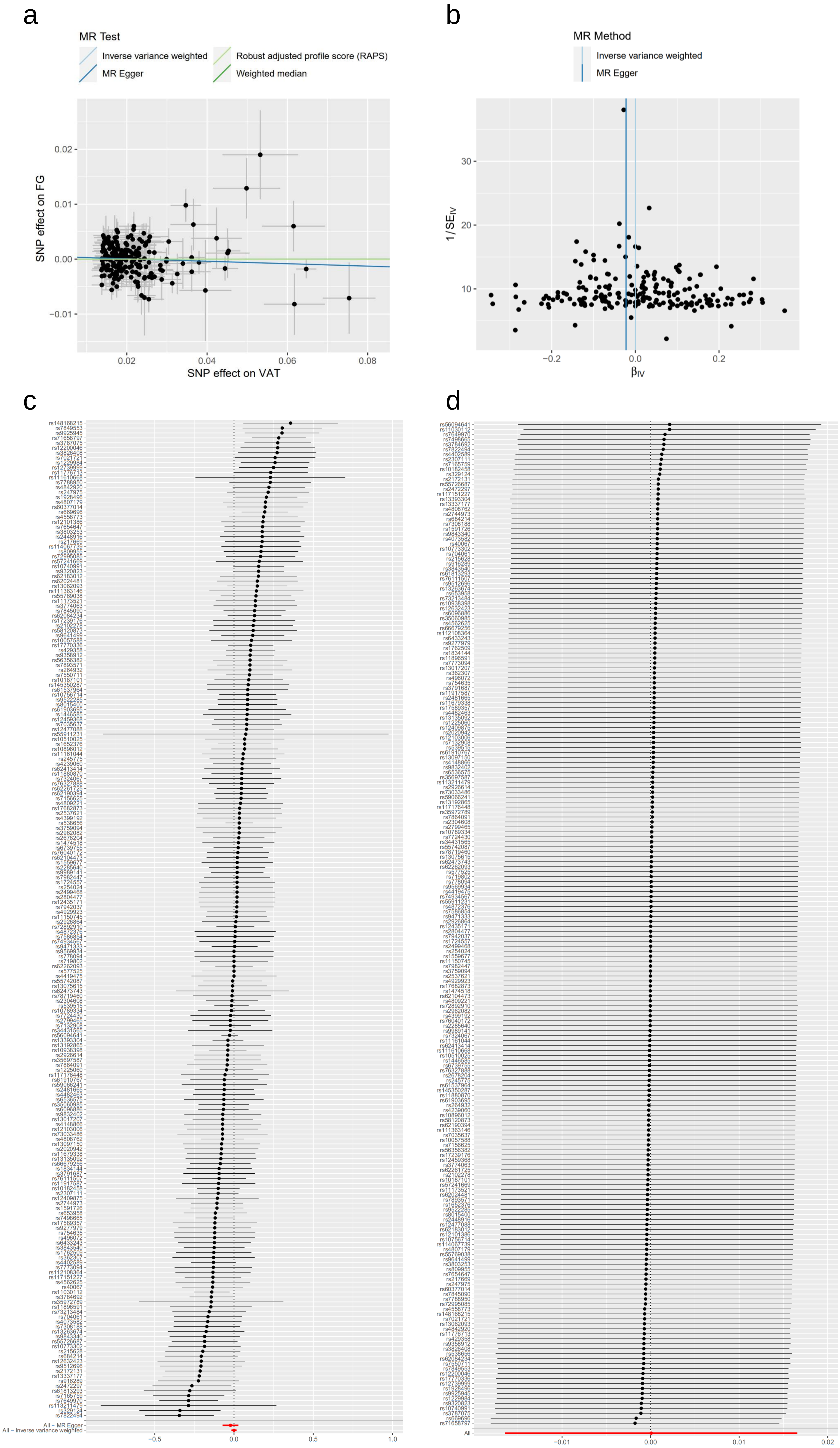

ESM Fig. 5. Scatter plot (a), funnel plot (b), forest plot (c), and leave-one-out test (d) for VAT on fasting glucose.

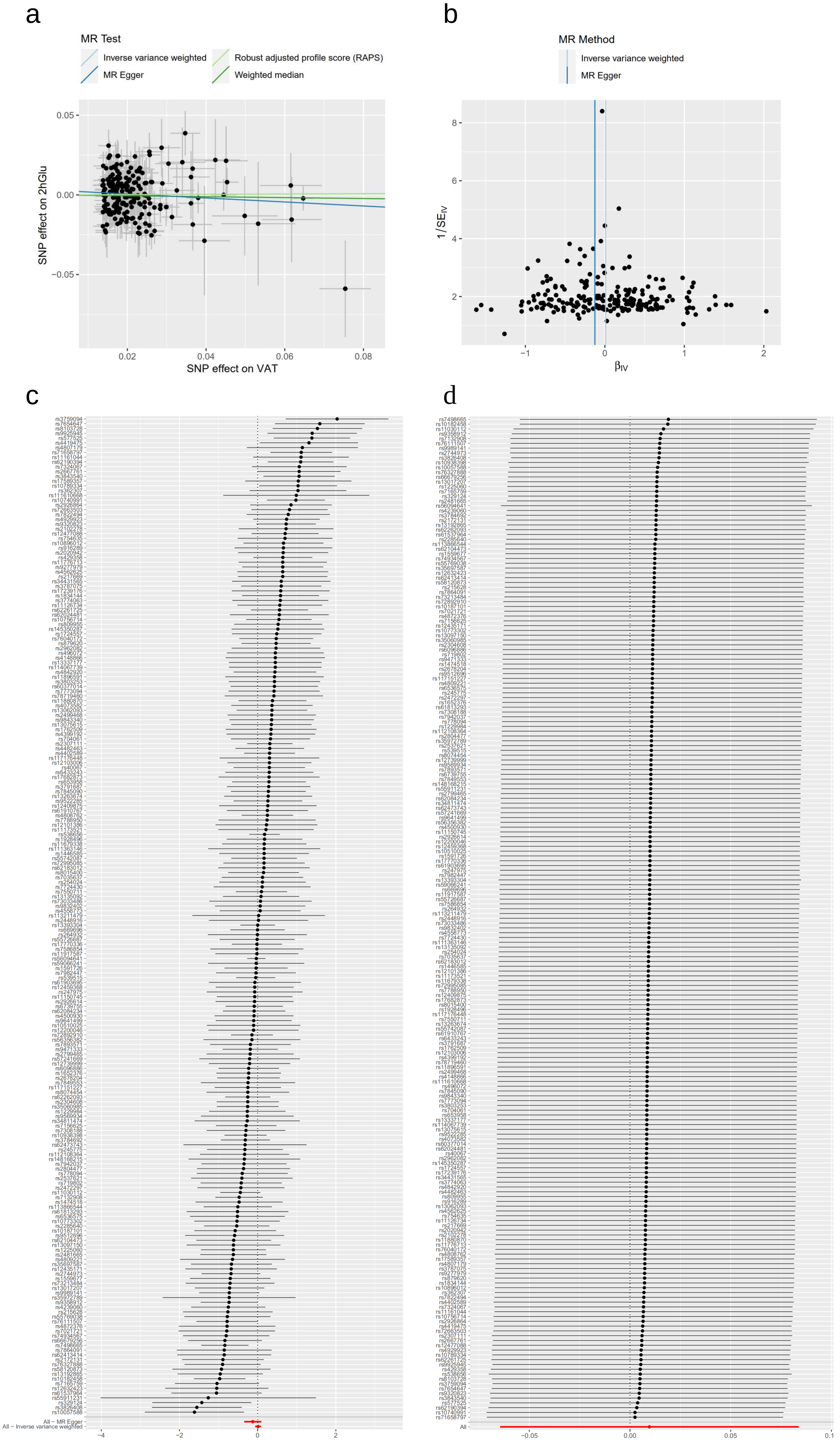

ESM Fig. 6. Scatter plot (a), funnel plot (b), forest plot (c), and leave-one-out test (d) for VAT on 2h-glucose.

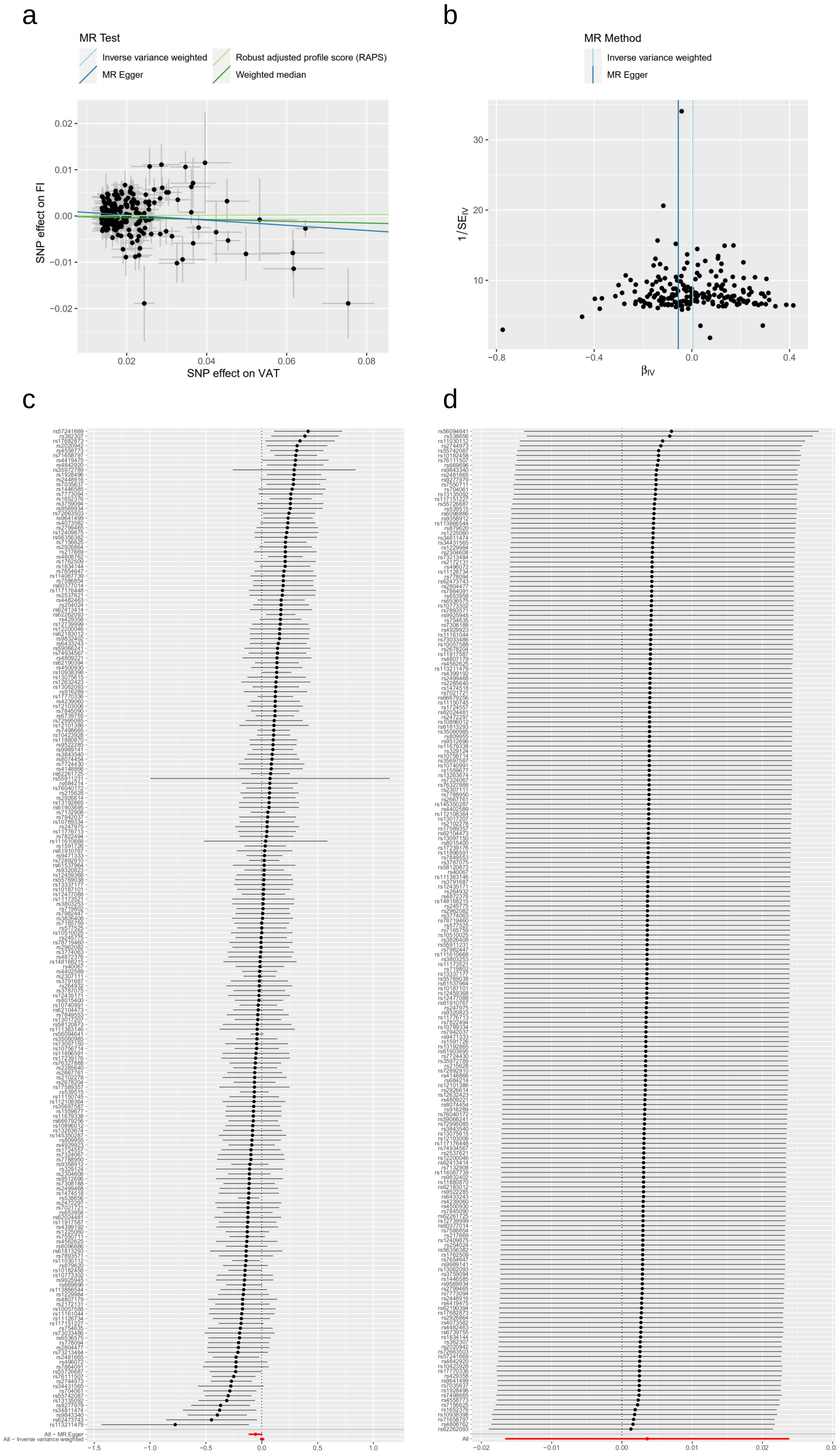

ESM Fig. 7. Scatter plot (a), funnel plot (b), forest plot (c), and leave-one-out test (d) for VAT on fasting insulin.

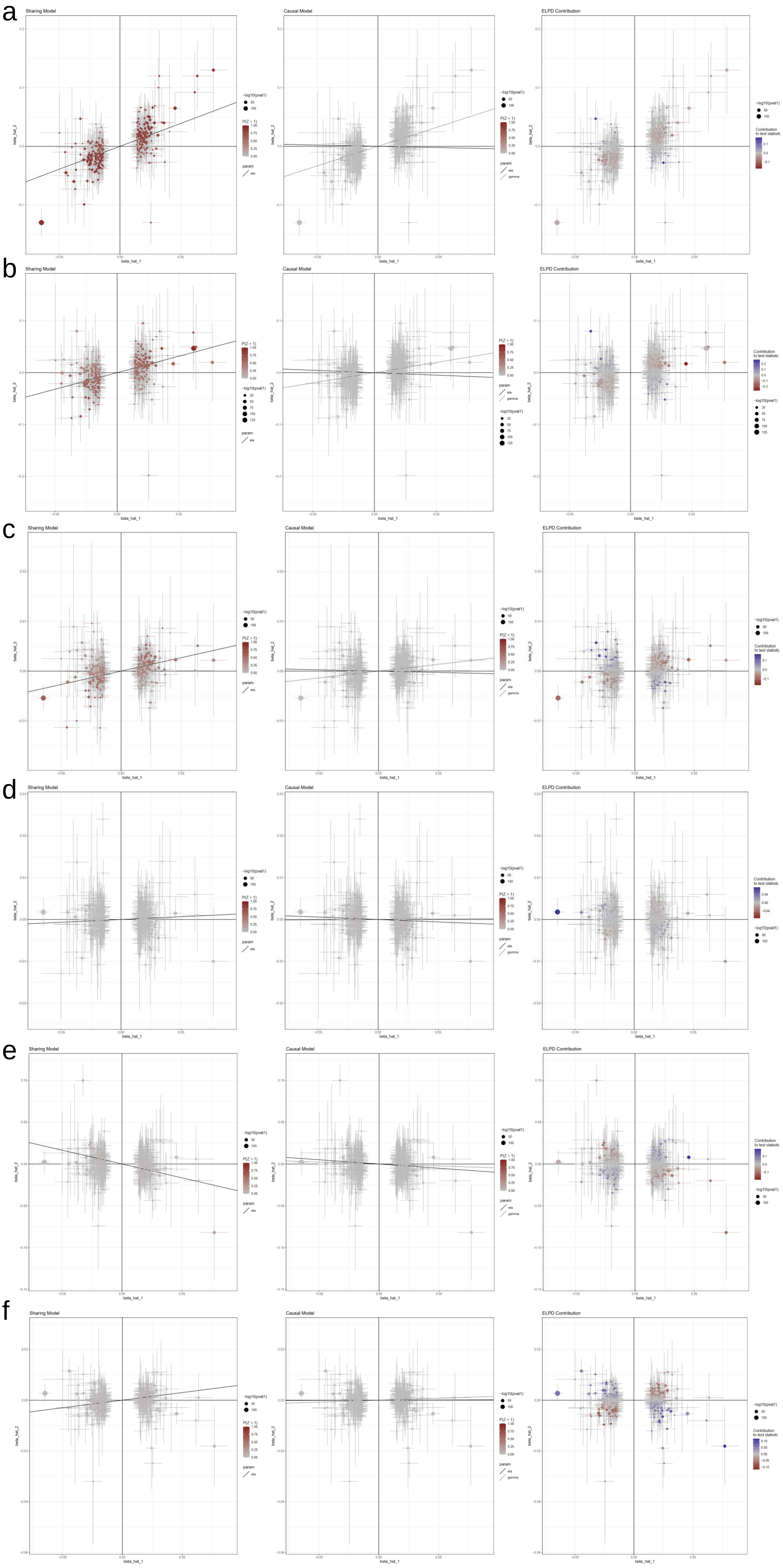

ESM Fig. 8 Scatter plot for discovery T2D (a), replication T2D (b), HbA1c (c), FG (d), 2hGlu (e), and FI (f)using CAUSE.

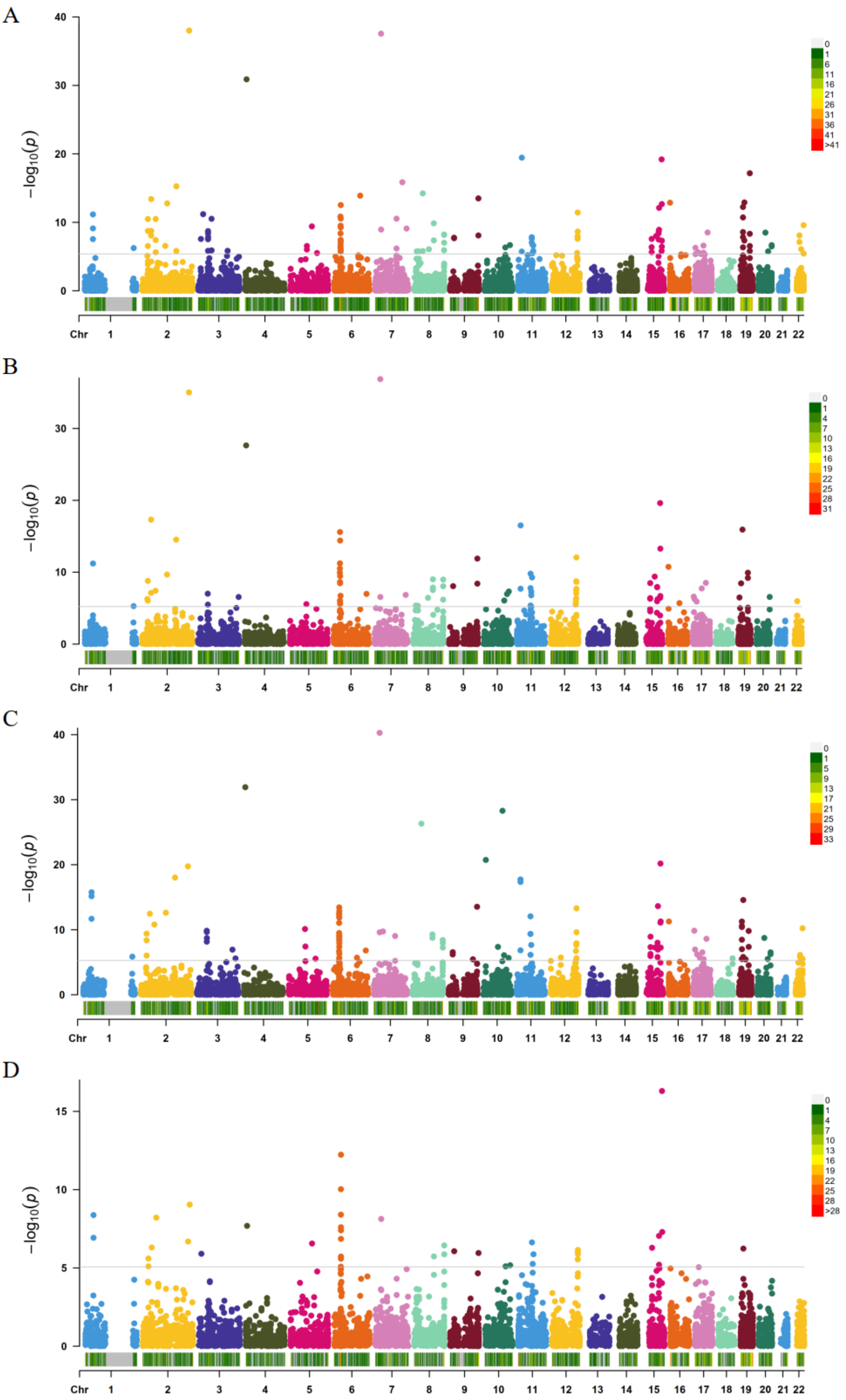

ESM Fig.9. Manhattan plots of JTI (a), PrediXcan (b), and UTMOST (c) and SMR (d). The results showing *P*-values on the -log10 scale on the y-axis for VAT gene expression-T2D associations. The grey horizontal line represents the relative Bonferroni corrected *P*-value.

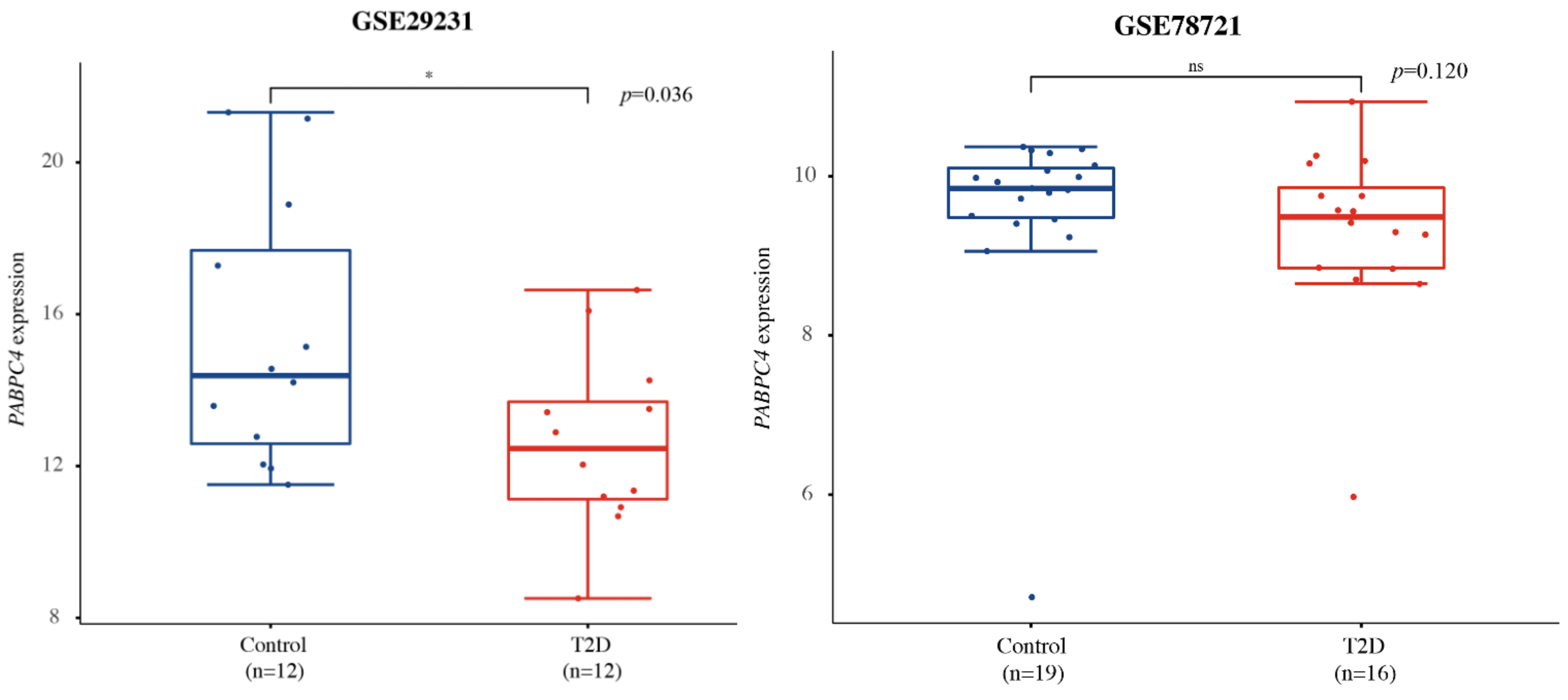

ESM Fig. 10. *PABPC4* expression in different publicly available data for visceral adipose.

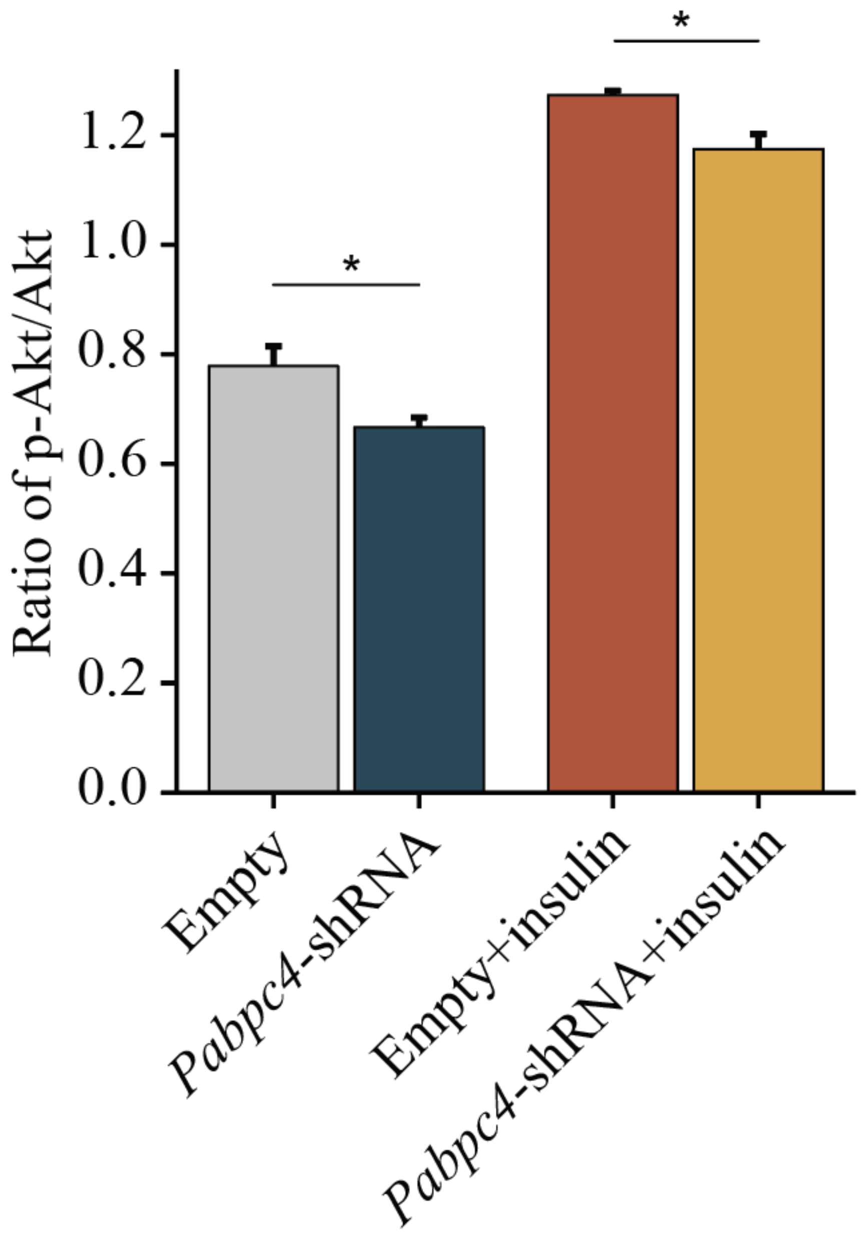

ESM Fig 11. P-akt/akt assessment.

[illegible]
